# Support Pore Structure and Composition Strongly Influence the Direct Air Capture of CO_2_ on Supported Amines

**DOI:** 10.1021/jacs.2c12707

**Published:** 2023-03-27

**Authors:** Guanhe Rim, Pranjali Priyadarshini, MinGyu Song, Yuxiang Wang, Andrew Bai, Matthew J. Realff, Ryan P. Lively, Christopher W. Jones

**Affiliations:** School of Chemical & Biomolecular Engineering, Georgia Institute of Technology, 311 Ferst Dr., Atlanta, Georgia 30332-0100, United States

## Abstract

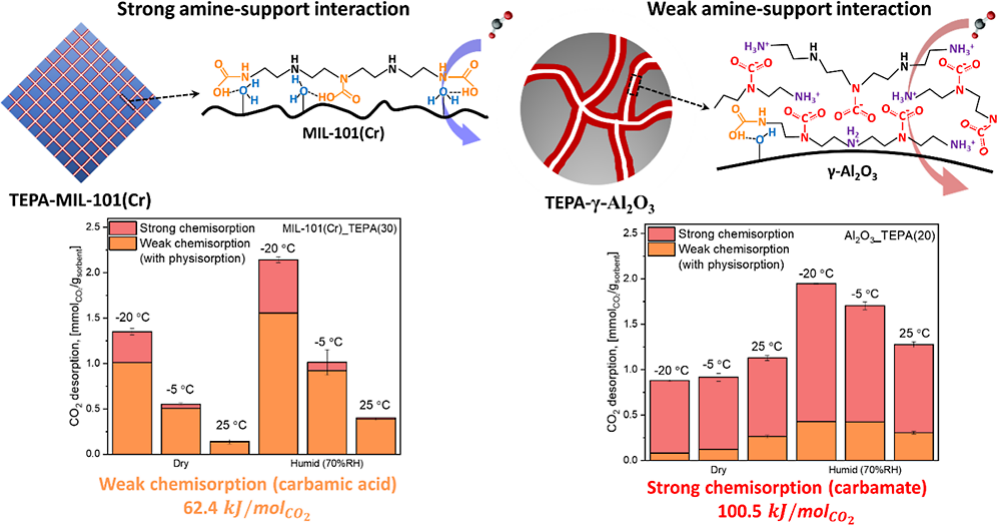

A variety of amine-impregnated porous solid sorbents for direct air capture (DAC) of CO_2_ have been developed, yet the effect of amine-solid support interactions on the CO_2_ adsorption behavior is still poorly understood. When tetraethylenepentamine (TEPA) is impregnated on two different supports, commercial γ-Al_2_O_3_ and MIL-101(Cr), they show different trends in CO_2_ sorption when the temperature (−20 to 25 °C) and humidity (0–70% RH) of the simulated air stream are varied. *In situ* IR spectroscopy is used to probe the mechanism of CO_2_ sorption on the two supported amine materials, with weak chemisorption (formation of carbamic acid) being the dominant pathway over MIL-101(Cr)-supported TEPA and strong chemisorption (formation of carbamate) occurring over γ-Al_2_O_3_-supported TEPA. Formation of both carbamic acid and carbamate species is enhanced over the supported TEPA materials under humid conditions, with the most significant enhancement observed at −20 °C. However, while equilibrium H_2_O sorption is high at cold temperatures (e.g., −20 °C), the effect of humidity on a practical cyclic DAC process is expected to be minimal due to slow H_2_O uptake kinetics. This work suggests that the CO_2_ capture mechanisms of impregnated amines can be controlled by adjusting the degree of amine-solid support interaction and that H_2_O adsorption behavior is strongly affected by the properties of the support materials. Thus, proper selection of solid support materials for amine impregnation will be important for achieving optimized DAC performance under varied deployment conditions, such as cold (e.g., −20 °C) or ambient temperature (e.g., 25 °C) operations.

## Introduction

Fast decarbonization of the energy sector requires the deployment of avoided emission technologies like carbon capture and sequestration from point sources as well as the deployment of negative emission technologies (NETs) such as direct air capture (DAC) coupled with carbon storage.^1^ DAC has several advantages over other NETs, such as bioenergy with carbon capture and storage,^2^ due to its lower requirement for land and water utilization.^3^ However, DAC must overcome challenges associated with high capital costs and moderate energy costs.

DAC has been intensively studied in recent years, and a variety of adsorbent materials have been developed to directly capture CO_2_ from the air. Among them, amine-functionalized porous materials are attractive adsorbents for DAC and are widely studied due to their promising performance under humid conditions, lower amine volatilization rates compared to amine solvents, and the strong affinity of amine groups for CO_2_.^4−6^ However, due to the high CO_2_ binding energy of amines, amine-based adsorbent materials require higher energy for CO_2_ desorption compared to physisorbents such as zeolites and MOFs. While the amine-containing solid adsorbents are tolerant to moisture in the air, it is generally known that physisorbents are not effective adsorbents for CO_2_ capture in the presence of moisture due to competitive adsorption between CO_2_ and H_2_O, which yields high sorbent regeneration costs. Thus, to utilize physisorbents for DAC, a two-stage process for the dehydration of air will likely be required.^7^

The behavior of solid-supported amines can be impacted by the structure and composition of the support materials because the porous structure of the support impacts amine dispersion and thus the access of CO_2_ to the amine sites.^5^ Moreover, differences in surface chemistries in support materials can result in different binding motifs between the amine and the support, which also has the potential to influence the CO_2_ capture performance. A variety of porous support materials have been used for amine impregnation, including silica,^8−40^ γ-alumina,^41−43^ MOFs,^44−46^ zeolite,^47^ and mixed metal oxides.^48,49^ Despite this, the effects of amine-solid support interactions on CO_2_ adsorption behavior are still poorly understood. Furthermore, most prior DAC studies are limited to adsorption temperatures above indoor ambient room temperature (>20 °C). Only our previous study on amine-impregnated MIL-101(Cr) reported detailed DAC behavior under sub-ambient temperature conditions (from −20 to 25 °C), including both dry^35^ and humid gases.^44^ Since there will likely be no optimal sorbent covering all conditions, including day/night, summer/winter, and rainy/dry conditions, designing and operating a DAC process will be complex. A high-performing adsorbent will, therefore, be a sorbent that operates well under all conditions, rather than one that is optimized for a narrow set of conditions. To this end, investigating the DAC performance of sorbent materials under varied conditions is crucial for the practical deployment of DAC technologies.^50^

As noted earlier, the adsorption mechanism (e.g., physisorption, the formation of carbamic acid, the formation of ammonium carbamate ion pairs, and the formation of ammonium bicarbonate) may vary depending on the degree of interaction between the impregnated amines and support. For example, specific molecular interactions between amines and MOFs like Mg_2_(dobpdc) lead to unique CO_2_ adsorption mechanisms^51−57^ compared to more ill-defined systems based on MOFs and PEI.^58−61^ We hypothesize that the CO_2_ adsorption pathways for impregnated amines within different support materials and/or pore structures may differ depending on adsorption temperature and humidities. Thus, to design optimized adsorbents for DAC processes operating in an ever-changing environment (outdoor air),^50^ an in-depth understanding of the roles of support materials, temperature, humidity on CO_2_ adsorption kinetics, and thermodynamics is required.

Here, to systematically probe the effect of support materials on the DAC performance of impregnated amines [tetraethylenepentamine (TEPA)], amine-based solid sorbents were synthesized with two different porous solid supports, MIL-101(Cr) and γ-Al_2_O_3_. TEPA was used for amine impregnation because this chemical mainly consists of primary and secondary amines, which have a higher potential for CO_2_ adsorption (e.g., heat of CO_2_ adsorption = −80 to −110 kJ/mol_CO_2__) compared to tertiary amines (−20 kJ/mol_CO_2__),^62,63^ as well as shorter amine chains that likely result in enhanced CO_2_ kinetics relative to the branched poly(ethylenimine) typically employed, especially under cold temperature conditions. Commercial γ-Al_2_O_3_ was selected for comparison with MIL-101(Cr) supports since they are already well-investigated support materials for amine impregnation and currently offer a more accessible pathway for scale up and deployment than MOFs. The materials’ CO_2_ adsorption behavior under both dry and humid (70% RH) 400 ppm CO_2_ conditions over a range of adsorption temperatures (−20 to 25 °C) was probed by CO_2_/H_2_O temperature-programed desorption (TPD) experiments and *in situ* FT-IR spectroscopy. We demonstrate that the properties of the porous solid support materials, such as pore size, pore volume, surface area, and support composition, have a significant effect on the CO_2_ adsorption pathways of the impregnated amines. The results presented here suggest that proper porous support materials should be considered for amine impregnation to operate an optimized DAC process, depending on the sub-ambient to ambient temperature conditions and humidities anticipated.

## Experimental Section

### Material Synthesis

Synthesis of MIL-101(Cr) powders was conducted based on the literature.^46,64^ The detailed synthesis process is described in our previous work.^44^ The synthesized MOF powders were dried under a high vacuum (about 10 mTorr) at 150 °C overnight for further analysis and amine impregnation. The MIL-101(Cr) and γ-Al_2_O_3_ powders were physically impregnated with 30 and 20 wt % TEPA loadings, respectively, to achieve a similar extent of pore filling (about 25%). The detailed procedure of amine impregnation is described in the Supporting Information.

### Characterization

#### Nitrogen Physisorption

A surface area and porosity (SAP) system (autosorb iQ/Quantachrome) was used for N_2_ physisorption experiments at 77 K. About 100 mg of MIL-101(Cr) and γ-Al_2_O_3_ were activated at 150 and 110 °C, respectively, under a vacuum for 15 h before the measurement. For the TEPA-impregnated MIL-101(Cr) and γ-Al_2_O_3_ powder sorbents, the activation was conducted at 60 °C under a vacuum for 3 h. The BET surface area was estimated using the N_2_ physisorption data in the *P*/*P*_0_ range of 0.05–0.2. Pore volumes of the materials were determined based on the N_2_ physisorption at a partial pressure of 0.995 and normalized per gram of support (MIL-101(Cr) or γ-Al_2_O_3_) in the powder sorbents.

#### CO_2_ Breakthrough Experiments under Dry and Humid Conditions with 400 ppm CO_2_

A custom-built fixed bed reactor described in the literature^44^ was used to perform dry and humid 400 ppm CO_2_ breakthrough experiments with TEPA-impregnated MIL-101(Cr) and γ-Al_2_O_3_ under a wide range of temperatures (−20, −5, and 25 °C) and humidities (0–70% RH). About 50 mg (wet basis) of powder sorbents were loaded inside the 5 cm fixed bed (1/4″ stainless steel tube with 4 mm ID) using glass wool on both ends of the column. A more detailed procedure is described in Supporting Information. In this study, small amounts of powder sorbents (<50 mg) were tested inside a 1/4″ stainless steel fixed bed column under an extremely diluted CO_2_ gas stream (400 ppm CO_2_/N_2_). The bed was always immersed in liquid (50% water/50% ethylene glycol) continuously circulating inside the bath of chiller (Julabo CD-600F) for efficient removal of the heat generated during the 400 ppm CO_2_ breakthrough. To confirm an isothermal condition of the fixed bed system, the convective heat transfer coefficient, *h*, needed to keep the temperature of the powder sorbents less than 1 °C above the flowing simulated air was estimated. For example, the required *h* was estimated to be 2.36 × 10^–3^ W/m^2^/K for 0.01 W of the heat generation rate during 400 ppm CO_2_ adsorption with the TEPA-impregnated γ-Al_2_O_3_ in the fixed bed system (45 mg of the sorbent, 94 m^2^/g_sorbent_, −Δ*H* = 100 kJ/mol_CO_2__, and 2.64 × 10^–4^ mmol/g/s adsorption rate). The estimated value was much lower than the *h* of natural convection in the air (2.5–25 W/m^2^/K, produced by density differences of air),^65^ indicating that convection cooling by flowing adsorption gas is enough to maintain an isothermal condition due to the slow heat generation rate of CO_2_ adsorption under an extremely diluted CO_2_ gas stream (400 ppm CO_2_/N_2_). Thus, it was assumed that the temperature change of the fixed bed column is negligible during the 400 ppm CO_2_ adsorption.

### Measurement of Energy for Dry/Wet CO_2_ and H_2_O Desorption

The interaction strength between CO_2_ and the amine-impregnated MIL-101(Cr) and γ-Al_2_O_3_ powder sorbents are expected to be different under varied dry and humid adsorption temperature conditions. Thus, it is crucial to quantify the binding energy of dry and humid CO_2_ (also H_2_O) to estimate an energy requirement for sorbent regeneration. In this study, TPD experiments were performed with varied heating rates to directly evaluate the energy required for desorption of CO_2_ (dry and humid) and H_2_O from amine-impregnated MIL-101(Cr) and γ-Al_2_O_3_ powder sorbents.^66^ Detailed procedures are described in Supporting Information, including a definition of the desorption energy used in this work.

### *In Situ* Diffuse Reflectance Infrared Fourier Transform Spectroscopy

*In situ* FT-IR spectroscopy was performed on a Nicolet iS10 IR spectrometer with a low-temperature diffuse reflectance infrared Fourier transform spectroscopy (DRIFTS) cell (CHC-CHA-4, Harrick Scientific Products Inc.). For the dry and wet 400 ppm CO_2_ adsorption studies, a sample holder inside the DRIFTS cell chamber was filled with about 50 mg of sorbent powders. The sample was then activated at 60 °C under 40 sccm N_2_ flow for 3 h. After the sample temperature cooled to the adsorption temperature (−20 °C or 25 °C), the inlet gas flow was switched to dry or wet (70% RH) 400 ppm CO_2_/N_2_ (40 sccm). During the adsorption, spectra were collected with 64 scans at a resolution of 4 cm^–1^ every 5 min. The humidity for the wet gas was precisely controlled by a wet gas generator (WETSYS/SETARAM).

## Results and Discussion

### Characterization of Amine (TEPA)-Impregnated MIL-101(Cr) and γ-Al_2_O_3_

To investigate the effect of support material structure and composition on the CO_2_ capture behavior of impregnated amines under a similar extent of pore filling (about 25%), MIL-101(Cr) and γ-Al_2_O_3_ porous supports were physically impregnated with TEPA to 30 and 20 wt % loadings, respectively. Key physical properties, including pore volumes, pore sizes, and BET surface areas of the bare support and amine-loaded sorbent materials were determined from N_2_ physisorption isotherms (Figure S1), and the results are summarized in Table 1. The particle size of two support materials is also included. The mean particle size of γ-Al_2_O_3_ was estimated based on SEM images (Figure S2). The properties of the MIL-101(Cr) materials are comparable to the prior literature.^44,46^ MIL-101(Cr) has a smaller average pore size (2.4 nm) than γ-Al_2_O_3_ (16.1 nm), with a much higher pore volume and BET surface area, suggesting that impregnated amines may interact with MIL-101(Cr) more significantly than γ-Al_2_O_3_ at similar loadings. Table 1 shows the extent of pore filling of sorbents determined based on the N_2_ physisorption isotherms of the bare support materials and amine-impregnated powder sorbents (Figure S1). The theoretical extent of pore filling was also calculated with an assumption that all TEPA was impregnated inside the pores of the support materials. The 30 wt % TEPA-impregnated MIL-101(Cr) [MIL-101(Cr)_TEPA(30)] and 20 wt % TEPA-impregnated γ-Al_2_O_3_ [Al_2_O_3__TEPA(20)] show almost identical extents of pore filling (25.4–27.6%). Close values between the measured and theoretical pore filling indicate that TEPA was effectively impregnated inside the pores of both MIL-101(Cr) and γ-Al_2_O_3_.

**Table 1:** Physical Properties of Support Materials and Adsorbents

support materials	MIL-101(Cr)	γ-Al_2_O_3_
particle size, [μm]	∼0.5^44^	6.9^a^
pore volume, [cc/g]	1.84	0.95
pore size (mode), [nm]	2.4	16.1
BET surface area, [m^2^/g]	3270	153
amine (TEPA) loadings, [wt %]	30	20
**powder sorbents**	MIL-101(Cr)**_TEPA**(30)	**Al**_**2**_**O**_**3**_**_TEPA**(20)
pore filling, [ %]	25.4 (by N_2_ physisorption)	27.6 (by N_2_ physisorption)
	23.3 (by calculation)	26.5 (by calculation)

a Mean particle size was determined based on SEM images (Figure S2).

### Humid and Dry CO_2_ Adsorption Behavior of Amine (TEPA)-Impregnated MIL-101(Cr) and γ-Al_2_O_3_ at Ambient and Cold Temperatures

**Figure 1:**
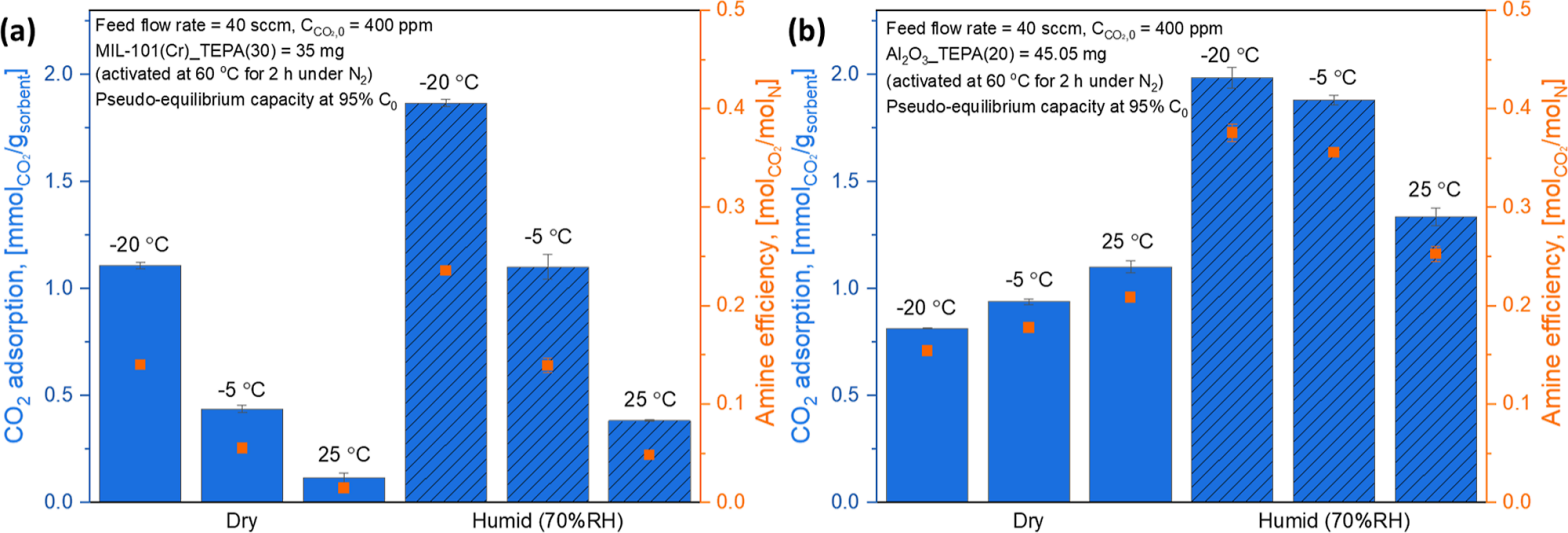
Pseudo-equilibrium 400 ppm CO_2_ adsorption capacities and amine efficiencies of (a) 30 wt % TEPA-impregnated MIL-101(Cr) and (b) 20 wt % TEPA-impregnated γ-Al_2_O_3_ powder adsorbents under dry and humid (70% RH) conditions at −20, −5, and 25 °C. Both materials have similar pore filling with TEPA.

To study the effects of temperature and humidity on the CO_2_ adsorption behavior of impregnated amines (TEPA) within different porous support materials, dry and humid (70% RH) 400 ppm CO_2_ breakthrough experiments were conducted with MIL-101(Cr)_TEPA(30) and Al_2_O_3__TEPA(20) powder sorbents at −20, −5, and 25 °C. Figure S3 shows the dry and humid (70% RH) CO_2_ breakthrough curves of the powder sorbents under different temperature conditions. Pseudo-equilibrium 400 ppm CO_2_ adsorption capacities of MIL-101(Cr)_TEPA(30) and Al_2_O_3__TEPA(20) were determined based on the breakthrough curves, and the results are shown in Figure 1a,b, respectively, with amine efficiencies.

The 400 ppm CO_2_ uptake of MIL-101(Cr)_TEPA(30) under dry conditions was significantly enhanced with decreasing temperature, from 0.11 mmol/g (25 °C) to 1.11 mmol/g (−20 °C) because the overall CO_2_ capture capacities are dominantly affected by thermodynamics rather than mass transfer due to the low degree of pore filling (25.4%, see Table 1).^44^ On the other hand, TEPA in γ-Al_2_O_3_ showed a different trend. The 400 ppm CO_2_ uptake of Al_2_O_3__TEPA(20) (1.10 mmol/g) at 25 °C gradually reduced with decreasing temperature down to −20 °C (0.81 mmol/g) even with a much larger pore size (16.1 nm > 2.4 nm) and similar extent of pore filling compared to MIL-101(Cr)_TEPA(30) (see Table 1). Interestingly, at −20 °C, the MIL-101(Cr)_TEPA(30) showed higher 400 ppm CO_2_ uptake (1.11 mmol/g) with lower amine efficiency (0.14) than Al_2_O_3__TEPA(20) (0.81 mmol/g with 0.15 amine efficiency), suggesting that a larger amount of CO_2_ physisorption probably occurred in the CO_2_ capture by the MIL-101(Cr)_TEPA(30) due to the smaller pore size of MIL-101(Cr) than γ-Al_2_O_3_ (2.4 nm < 16.1 nm).

Under humid conditions, the overall DAC performances of the two powder sorbents were enhanced. Similar observations for amine sorbents in the presence of humidity have been attributed to more carbamate/carbamic acid species forming due to the freeing of amines from support interactions^17,67,68^ or the formation of bicarbonate under humid conditions.^69^ The form(s) of captured CO_2_ under dry and humid conditions are further discussed below with insight from *in situ* FT-IR experiments. Moreover, MIL-101(Cr)_TEPA(30) was shown to be more significantly affected by the presence of humidity than Al_2_O_3__TEPA(20). These different CO_2_ capture behaviors of the MIL-101(Cr)_TEPA(30) and Al_2_O_3__TEPA(20) under dry and humid conditions strongly suggest that the local environment of TEPA within each support is different. To further probe the impact of the support materials (or pore structures) on the CO_2_ adsorption mechanism(s) of the impregnated amine (TEPA), CO_2_/H_2_O temperature-programed desorption (TPD) and *in situ* FT-IR experiments were conducted.

**Figure 2:**
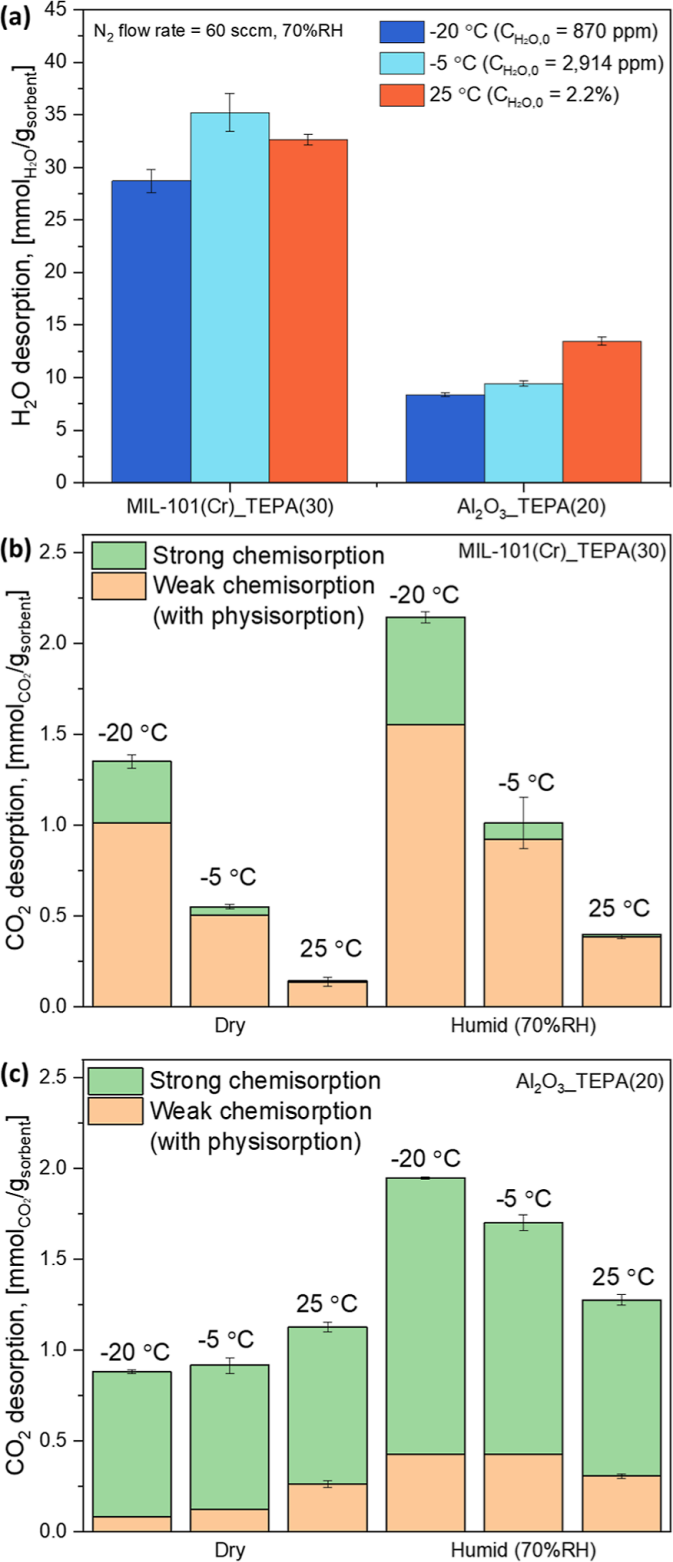
(a) Amount of desorbed H_2_O from TEPA-impregnated MIL-101(Cr) and γ-Al_2_O_3_ powder adsorbents during the CO_2_/H_2_O TPD. Amount of desorbed dry/humid CO_2_ from (b) 30 wt % TEPA-impregnated MIL-101(Cr) and (c) 20 wt % TEPA-impregnated γ-Al_2_O_3_ powder adsorbents during the CO_2_/H_2_O TPD. Strong chemisorption: CO_2_ desorption above 25 °C; weak chemisorption: CO_2_ desorption under 25 °C.

Representative CO_2_/H_2_O TPD experimental results are shown in Figure S4. The desorbed CO_2_ and H_2_O during the TPD experiments were quantified as a function of time (Figure S5). The total amounts of desorbed H_2_O from the MIL-101(Cr)_TEPA(30) and Al_2_O_3__TEPA(20) materials after humid (70% RH) 400 ppm CO_2_ breakthroughs are plotted in Figure 2a as a function of adsorption temperature. Since the humid CO_2_ capture experiments were conducted at constant relative humidity (RH) conditions, 70% RH, under varied adsorption temperatures from −20 to 25 °C, the absolute humidity range was very wide (870 ppm–2.2% by volume). As a result, we observed that the H_2_O uptake by MIL-101(Cr)_TEPA(30) did not consistently change with adsorption temperature conditions. While the H_2_O uptake slightly increased from 25 °C (32.7 mmol/g) to −5 °C (35.2 mmol/g), it decreased at −20 °C (28.7 mmol/g), likely due to the extremely low absolute humidity (870 ppm) at this temperature. Overall, these uptake values are consistent with liquid-like conditions in the pores of the MIL-101(Cr). In the case of Al_2_O_3__TEPA(20), the H_2_O sorption continuously decreased with decreasing adsorption temperature, from 25 °C (13.5 mmol/g) to −20 °C (8.3 mmol/g).

Interestingly, the MIL-101(Cr)_TEPA(30) showed significantly higher H_2_O uptake than the Al_2_O_3__TEPA(20) under all temperature conditions. This is likely due to the much smaller pore size of MIL-101(Cr) (2.4 nm) than γ-Al_2_O_3_ (16.1 nm) (Table 1), yielding higher capillary condensation at lower partial pressures of water, as shown in Figure S6. It was reported that the process of capillary condensation of MIL-101(Cr) begins at 35% RH at 25 °C,^70^ while γ-Al_2_O_3_ begins at 65% RH at 22–25 °C,^71,72^ which is consistent with measured water vapor adsorption isotherm in this study (Figure S6). When compared to the humid 400 ppm CO_2_ adsorption results shown in Figure 1a,b, one can see that the H_2_O uptake is more significant during the humid DAC process at all temperature conditions. Thus, assuming similar Δ*H*_ads(H_2_0)_ for these materials, more energy will be required for the regeneration of MIL-101(Cr)_TEPA(30) than for Al_2_O_3__TEPA(20) for both ambient and cold humid DAC processes due to the significant H_2_O uptake. However, it should be noted that the H_2_O uptake kinetics are extremely slow at cold temperature conditions due to the low absolute humidity and the effect of temperature on the overall kinetics (Figure S7). Thus, if water sorption is kinetically limited, regeneration energies can be significantly reduced.

In this study, weak chemisorption (including physisorption) is defined as CO_2_ desorption below 25 °C and strong chemisorption is defined as CO_2_ desorption above 25 °C, as discussed in Supporting Information (Figure S8). The amounts of weak and strong chemisorption were quantified based on Figure S5 and the results are plotted in Figure 2b,c for the MIL-101(Cr)_TEPA(30) and Al_2_O_3__TEPA(20), respectively. The figures clearly show that the impregnated TEPA within MIL-101(Cr) and γ-Al_2_O_3_ exhibit different CO_2_-amine interactions. Inside smaller pores (2.4 nm) with higher remaining pore volume (1.84 cc/g), as in MIL-101(Cr), TEPA showed weak chemisorption dominant behaviors.^44^ Inside a larger pore (16.1 nm) support with lower pore volume (1.03 cc/g), as in γ-Al_2_O_3_, strong chemisorption was the dominant CO_2_ capture mechanism of TEPA. Both weak and strong chemisorption was enhanced under humid conditions.

### Effect of Support Materials on the CO_2_ Adsorption Mechanism(s) of Impregnated Amines (TEPA) under Dry Conditions

**Figure 3:**
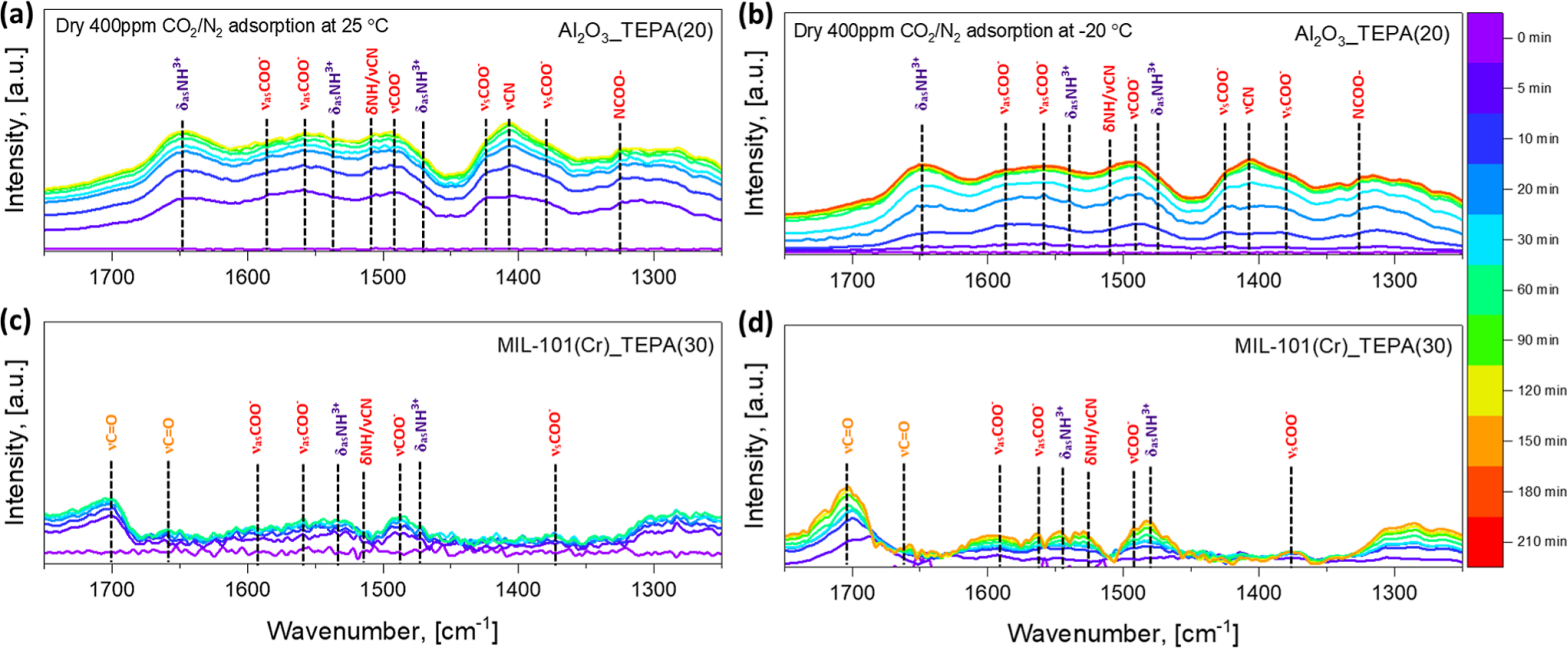
*In situ* FT-IR spectra of (a/b) 20 wt % TEPA-impregnated γ-Al_2_O_3_ and (c/d) 30 wt % TEPA-impregnated MIL-101(Cr) powder adsorbents as a function of adsorption time at (a/c) 25 °C and (b/d) −20 °C with the activated sample as the background. Orange: carbamic acid, red: carbamate ion, purple: ammonium ion. Adsorption conditions: gas, 400 ppm CO_2_/N_2_; flow rate, 40 sccm; relative humidity, dry; activation, 60 °C under 40 sccm N_2_ for 2–3 h.

To identify the adsorbed CO_2_ species associated with weak and strong chemisorption, *in situ* FT-IR experiments were conducted at 25 and −20 °C under dry 400 ppm CO_2_ environments (Figure 3). It should be noted that these spectra were obtained after subtracting the activated sorbent spectra from the IR spectra after a given time of exposure to dry 400 ppm CO_2_ to observe contributions more clearly from CO_2_ adsorption. The relevant peak assignments are summarized in Table S1 with supporting references.

Results shown in Figure 3a,c indicate that the dominant adsorbed CO_2_ species on the Al_2_O_3__TEPA(20) and MIL-101(Cr)_TEPA(30) at 25 °C are different. The TEPA-impregnated MIL-101(Cr) appears to dominantly form carbamic acid with a small amount of ammonium carbamate ion pairs, while the TEPA-impregnated γ-Al_2_O_3_ adsorbents only or primarily form ammonium carbamate ion pairs at the same conditions. It is known that carbamate ion species (strong chemisorption) have higher binding energies than carbamic acid species (weak chemisorption),^73,74^ and the form of adsorbed CO_2_ here correlates well with the ease of desorption, as determined by the TPD experiments.

The measured FT-IR spectra of Al_2_O_3__TEPA(20) and MIL-101(Cr)_TEPA(30) under dry 400 ppm CO_2_ flow at −20 °C (sub-ambient) are shown in Figure 3b,d, respectively. It appears that the same adsorbed CO_2_ species formed at 25 °C were also formed at −20 °C on each sorbent, while there are differences in the CO_2_ adsorption kinetics and the total amount of adsorbed CO_2_ species at equilibrium under the different temperature conditions. Obvious increases in the intensity of adsorbed CO_2_ in both adsorbents were observed as a function of time at 25 °C, whereas the intensities relatively slowly increased at −20 °C, indicating reduced CO_2_ adsorption kinetics at cold temperature conditions, as also observed in breakthrough experiments described above. The overall intensity of adsorbed CO_2_ on Al_2_O_3__TEPA(20) at equilibrium slightly decreased from 25 to −20 °C, suggesting that the formation of ammonium carbamate ion pairs may be suppressed at sub-ambient temperature conditions. Given that carbamate formation requires cooperative interaction of two amine sites, lower amine chain mobility at low temperatures may impede carbamate formation. In the case of carbamic acid formation on the MIL-101(Cr)_TEPA(30) powder sorbents, sharper and higher equilibrium intensities were observed at −20 °C, even with reduced CO_2_ adsorption kinetics, indicating the enhanced formation of carbamic acid. These *in situ* FT-IR results are consistent with the CO_2_ desorption data shown in Figure 2b,c. The effects of temperature on the formation of carbamic acid and ammonium carbamate ion pairs will be further discussed below.

Also noteworthy is that an apparent physisorbed CO_2_ peak at 2335 cm^–1^ was observed on the MIL-101(Cr)_TEPA(30) at −20 °C, while the Al_2_O_3__TEPA(20) did not show any distinct CO_2_ physisorption peak, as depicted in Figure S9. Thus, it can be inferred that the higher pseudo-equilibrium 400 ppm CO_2_ uptake of MIL-101(Cr)_TEPA(30) vs Al_2_O_3__TEPA(20) with lower amine efficiency at −20 °C (Figure 1) is due to a relatively larger amount of CO_2_ physisorption over MIL-101(Cr)_TEPA(30). We infer that TEPA impregnation inside the small pores of MIL-101(Cr) (2.4 nm) does two things: (i) it leaves relatively isolated primary amines leading to carbamic acid formation, and (ii) it leaves behind suitable micropore space for physisorption of CO_2_.

**Figure 4:**
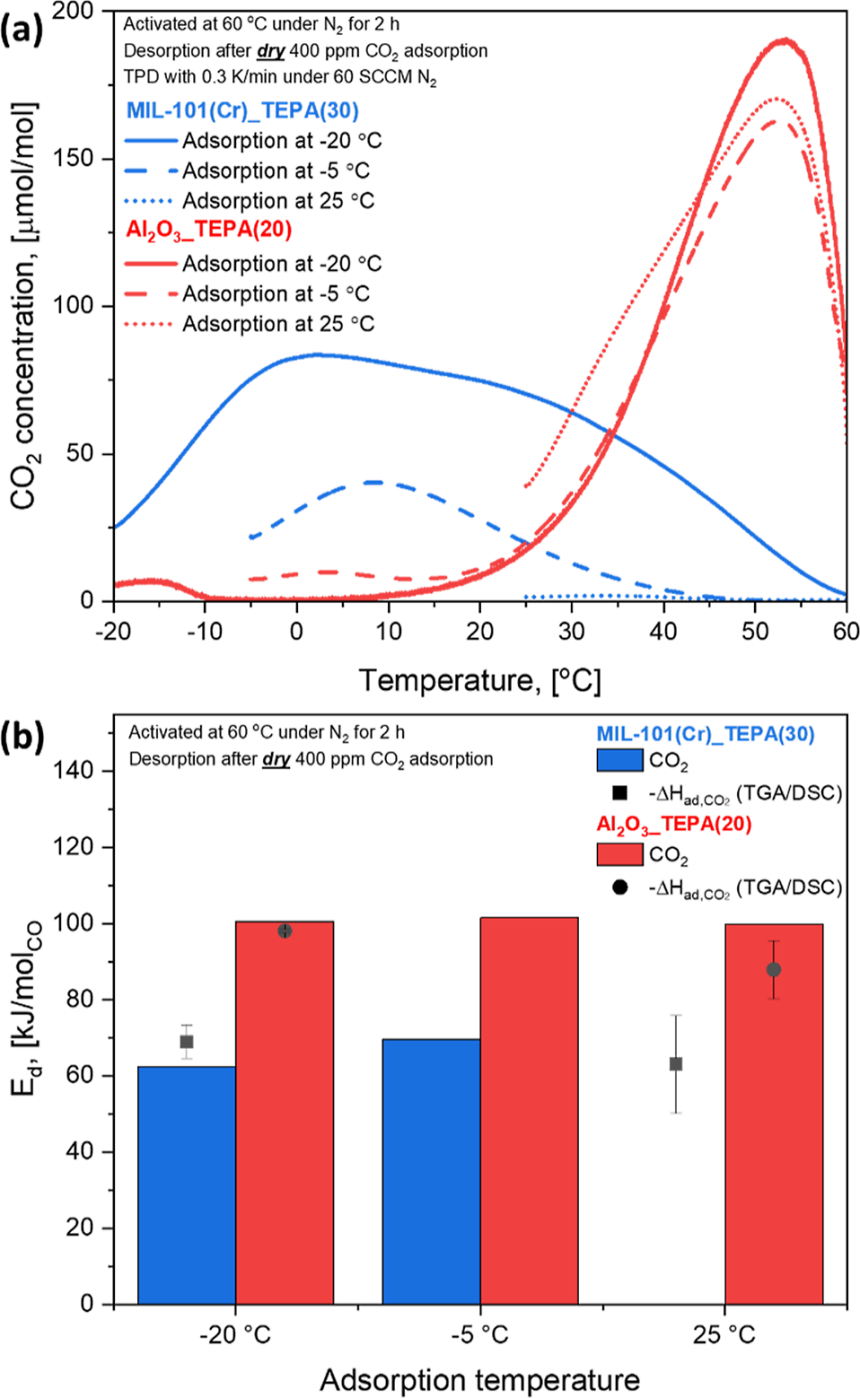
(a) CO_2_ TPD profiles and (b) energy of CO_2_ desorption of 30 wt % TEPA-impregnated MIL-101(Cr) and 20 wt % TEPA-impregnated γ-Al_2_O_3_ powder adsorbents for dry 400 ppm adsorption at −20, −5, and 25 °C. Δ*H*_ad,CO_2__: measured heat of dry 400 ppm CO_2_ adsorption by TGA/DSC.

The dry CO_2_ TPD profiles of MIL-101(Cr)_TEPA(30) and Al_2_O_3__TEPA(20) shown in Figure 4a clearly show the difference in CO_2_ desorption trends (weak and strong chemisorption). A desorption peak of MIL-101(Cr)_TEPA(30) was shown at a relatively low temperature between 30 and 35 °C for the 25 °C adsorption temperature, and it was shifted to 2.2 °C when decreasing the adsorption temperature down to −20 °C, indicating that the weak chemisorption became more significant at colder adsorption temperatures.^44^ The *in situ* FTIR results shown in Figure 3 strongly indicate that the weak chemisorption is related to the formation of carbamic acid. In contrast, a desorption peak of Al_2_O_3__TEPA(20) was confirmed at a much higher temperature (53 °C), consistent with the strong chemisorption mechanism associated with formation of ammonium carbamate ion pairs (Figure 3).

Interestingly, the desorption peak position and the amount of desorbed CO_2_ (peak area) of the Al_2_O_3__TEPA(20) adsorbent were not significantly changed when decreasing the adsorption temperature, indicating that the formation of ammonium carbamate (a strongly exothermic reaction) is not significantly affected by the adsorption temperature, although lower temperatures enthalpically favor the adsorption process. On the other hand, the formation of carbamic acid (a less exothermic reaction) was enhanced at cold temperatures (Figure 4a). These different trends are probably due to the effects of temperature on amine mobility, in which amine mobility dramatically decreases with decreasing temperature conditions.^75^ The formation of ammonium carbamate ion pairs occurs via a reaction between two amine groups (primary or secondary) and CO_2_ with a 2:1 molar ratio, while the reaction ratio is 1:1 for the formation of carbamic acid. We hypothesize that the formation of the ammonium carbamate ion pairs may be more strongly impacted by the decrease in amine mobility at cold temperatures than the formation of carbamic acid due to the higher amine to CO_2_ reaction ratio (2 > 1). Thus, the expected large thermodynamic enhancement of the more exothermic carbamate ion pair formation is mitigated to some degree by arrested amine chain mobility. In contrast, chain motion is less important for carbamic acid formation, requiring only a single adsorption site, and thus, thermodynamic effects are more prominent. Thus, the MIL-101(Cr)_TEPA(30) and Al_2_O_3__TEPA(20) show different DAC behavior at ambient and sub-ambient temperature conditions (Figure 1).

The CO_2_ TPD results shown in Figure 4a indicate that the interaction strength between CO_2_ and the impregnated TEPA is different depending on the support materials (MIL-101(Cr) and γ-Al_2_O_3_) and adsorption temperature conditions. To quantify the binding energy of dry CO_2_ onto the adsorbent materials, CO_2_ TPD experiments were performed with varied heating rates and the energy of CO_2_ desorption, *E*_d_, was calculated based on the method described in the Supporting Information. The procedure is described in more detail in Figures S10 and S11. The heat of CO_2_ adsorption is an indicator of the strength of the interaction between the CO_2_ molecules and amine sites on sorbents, and it is released as heat during the exothermic CO_2_ adsorption process. The total energy of CO_2_ desorption, as defined in this work, is the energy required to overcome the thermodynamic barrier for desorption (Δ*H*_ads_) + the energy needed to overcome any kinetic barrier to desorption (EA_des_). Sensible heats are typically small under these conditions and are therefore often neglected.

The results are plotted in Figure 4b for MIL-101(Cr)_TEPA(30) and Al_2_O_3__TEPA(20), as a function of adsorption temperature. The *E*_d_ of MIL-101(Cr)_TEPA(30) for 25 °C adsorption was not determined because most of captured CO_2_ was desorbed during the N_2_ purging period at 25 °C (Figure S5e). The heat of dry 400 ppm CO_2_ adsorption, as measured *in situ* by TGA/DSC, is also plotted. Since the energy required for the desorption process (*E*_d_) is comparable to the heat released during the adsorption process (Δ*H*_ads_), the kinetic barrier for desorption (e.g., activation energy, EA_des_) is very small or negligible, and thus, the desorption energy determined by the TPD method approximates the heat of adsorption.^76^ The *E*_d_ of MIL-101(Cr)_TEPA(30) was slightly reduced (69.6 kJ/mol_CO_2__ to 62.4 kJ/mol_CO_2__) with decreasing the adsorption temperature from −5 to −20 °C because of enhanced weak chemisorption (or formation of carbamic acid) at cold temperature conditions (Figure 4a). In contrast, the CO_2_ desorption peak of the Al_2_O_3__TEPA(20) was not shifted under different adsorption temperature conditions (Figure 4a), resulting in a similar *E*_d_ (99.9–101.5 kJ/mol_CO_2__), consistent with strong chemisorption (or formation of ammonium carbamate pairs) at all adsorption temperature conditions, assuming ideal mixing (i.e., , the sorption enthalpy). These results clearly show that the impregnated TEPA has different CO_2_ capture mechanisms (carbamic acid vs carbamate) within different support materials, and the ammonium carbamate pair (100.5 kJ/mol_CO_2__) has stronger binding energy than carbamic acid (62.4 kJ/mol_CO_2__).^74,77,78^ The regeneration of MIL-101(Cr)_TEPA(30) and Al_2_O_3__TEPA(20) saturated with dry 400 ppm CO_2_ at −20 °C was monitored with *in situ* FTIR, and the results shown in Figure S12 support the assertion that carbamate species are stronger chemisorption products than carbamic acid.

### CO_2_ Adsorption Via Carbamic Acid Versus Ammonium Carbamate Ion Pair Formation

The abovementioned results are consistent with the previous work of Bacsik and Hedin (2011), who previously elucidated the formation of carbamate species vs carbamic acid species over amine-based sorbents.^79,80^ Their *in situ* FT-IR study revealed that the relatively low amine-loaded silica (AMS-6) adsorbents captured CO_2_ by forming both carbamic acid and ammonium carbamate pairs, while the high amine-loaded silica primarily formed ammonium carbamate ion pairs during CO_2_ adsorption (Figure S13a). Another work by Didas et al. (2014) showed similar results (Figure S13b).^81^ Yoo et al. (2015)^73^ controlled the formation of carbamic acid and ammonium carbamate pairs when amines react with CO_2_ by functionalizing a mesoporous silica SBA-15 with single (MONO) and triaminosilanes (TRI), as shown in Figure S14. Since the length of the MONO molecules was estimated to be sufficient for the interaction between primary amines (−NH_2_) and silanols (−OH) on the surface of SBA-15 but not for interaction with other amines,^82,83^ the MONO grafted adsorbents captured CO_2_ via interaction between primary amines and surface silanols, forming carbamic acid species stabilized by surface silanols with low isosteric heats of adsorption (∼−46 kJ/mol).^73^ On the other hand, the TRI grafted adsorbents captured CO_2_ via intramolecular amine–amine interactions within a single chain, forming ammonium carbamate ion pairs that have higher heats of adsorption of ∼−73 to −78 kJ/mol than the MONO adsorbents. It can be inferred from this prior study that the formation of carbamic acid is probably dominant when the interaction between impregnated amines and support materials is strong. These studies indicate that enough surface silanols are required (strong amine-support material interactions) to form carbamic acid stabilized by surface silanol groups, while ammonium carbamate pairs are the dominant species of adsorbed CO_2_ when amine–amine interactions are strong via amine clustering.

In the same manner, one possible cause for the different CO_2_ adsorption mechanisms of impregnated TEPA within the two support materials studied here, MIL-101(Cr) and γ-Al_2_O_3_, could be the result of different extents of interaction between the impregnated amines and the support materials. As shown in Table 1, MIL-101(Cr) has a much higher BET surface area to pore volume ratio of 1.78 nm^–1^ than γ-Al_2_O_3_ (0.16 nm^–1^), indicating that an equivalent amount of impregnated amines (filling a similar pore volume percentage) may more strongly interact with MIL-101(Cr) than γ-Al_2_O_3_. This is consistent with the observation that the dominant CO_2_ adsorbed species on MIL-101(Cr)_TEPA(30) adsorbents were carbamic acids, while the Al_2_O_3__TEPA(20) primarily formed ammonium carbamate pairs during dry 400 ppm CO_2_ adsorption, as shown in Figure 3.

The empirical formula of MIL-101(Cr) is Cr_3_(O)OH(bdc)_3_(H_2_O), where bdc is benzene-1,4-dicarboxylate. The octahedral trinuclear Cr(III)_3_O building units of MIL-101(Cr) are saturated with one hydroxyl group and two water molecules. MIL-101(Cr) is generally activated at 150 °C under a high vacuum to remove the water molecules, creating potential Lewis acid sites.^84−86^ Since the MIL-101(Cr)_TEPA(30) was activated at 60 °C under 1 atm N_2_ flow, the open metal sites of MIL-101(Cr) saturated with water during the amine impregnation process with MeOH solution might be mostly occupied with water molecules even after the activation process due to the mild conditions used. The theoretical maximum amount of hydroxyl groups and water molecules on the surface of MIL-101(Cr) is 4.2 mmol OH and H_2_O/g MOFs. Thus, as shown in Figure S15a, the formed carbamic acid on the MIL-101(Cr)_TEPA(30) adsorbents might be stabilized by water molecules or hydroxyl groups occupying the open metal sites of MIL-101(Cr).

The Al_2_O_3__TEPA(20) may also contain hydroxyl groups on the surface of alumina because it was confirmed that traces of water are generated from γ-Al_2_O_3_ during heating, even after activation at 1000 °C.^87^ Hydroxylation of the alumina surface occurs by the dissociative adsorption of water to Al^3+^, where Lewis acid sites and residing protons on lattice oxygens form surface hydroxyl groups.^88^ The reported heat of water adsorption on γ-Al_2_O_3_ at 25 °C under low water coverage (0.4 H_2_O/nm^2^) is −158.8 kJ/mol,^71^ indicating that the activated Al_2_O_3__TEPA(20) at 60 °C under N_2_ flow certainly contains surface hydroxyl groups. Lagauche et al. also reported that the coverage of surface OH groups on the activated γ-Al_2_O_3_ at 100 °C for 4 h is about 11.7 OH/nm^2^ or 4.4 mmol OH/g alumina,^89^ which is comparable to the theoretical maximum of MIL-101(Cr). Although γ-Al_2_O_3_ has a similar amount of surface hydroxyl groups to MIL-101(Cr), the degree of interaction between impregnated amine groups and hydroxyl groups on the surface of alumina will be much lower than in the case of MIL-101(Cr) due to the much lower BET surface area to pore volume ratio of γ-Al_2_O_3_ (0.16 nm^–1^) than that of MIL-101(Cr) (1.78 nm^–1^), as discussed above. Thus, it can be inferred that ammonium carbamate pairs are dominantly formed via amine–amine interactions during the CO_2_ adsorption by Al_2_O_3__TEPA(20), probably due to the lower degree of interaction between impregnated TEPA and γ-Al_2_O_3_ (Figure S15c).

The amine-impregnated MIL-101(Cr) powder sorbents also adsorbed 400 ppm CO_2_ via the formation of ammonium carbamate pairs when amine loading was high enough for significant amine clustering to occur (50 wt % TEPA). As shown in Figure S16, the CO_2_ TPD^44^ and *in situ* FT-IR studies revealed that both strong (ammonium carbamate) and weak chemisorption (carbamic acid) are involved in the CO_2_ adsorption by high amine loaded MIL-101(Cr) adsorbents (Figure S15b), while the low amine loaded adsorbents only showed weak chemisorption (carbamic acid) dominant behavior at −20 °C (Figure S15a). It appears that the CO_2_ capture mechanisms of amine-impregnated porous solid sorbents, including the formation of carbamic acid (weak chemisorption) or ammonium carbamate (strong chemisorption), can be controlled by adjusting the extent of pore filling and interactions between amine and the surface of support materials. Thus, the selection of support materials for amine impregnation could be an important factor in determining the DAC performance of the amine-impregnated solid adsorbent materials.

### Effect of Moisture on CO_2_ Adsorption Behavior of Amine (TEPA) within Different Support Materials (MIL-101(Cr) and γ-Al_2_O_3_)

The dry/humid 400 ppm CO_2_ breakthrough experiment results described in Figure 1 clearly show that moisture has a positive effect on the DAC performance of the TEPA-impregnated MIL-101(Cr) and γ-Al_2_O_3_ powder sorbents at both ambient (25 °C) and sub-ambient (−5 and −20 °C) temperatures. Similar results were also reported in our previous work with the amine-impregnated MIL-101(Cr).^44^ To gain in-depth understanding of the effects of humidity, the CO_2_/H_2_O TPD profiles after humid (70% RH) CO_2_ adsorption are compared with the results of dry CO_2_ adsorption at different adsorption temperatures (25, −5, and −20 °C) in Figure S17. At the same time, the measured *in situ* FT-IR spectra (1750–1250 cm^–1^) during the humid 400 ppm CO_2_ adsorption (70% RH) at 25 and −20 °C are shown in Figure S18 to support the discussion of the effects of humidity. Figure S19 shows overall *in situ* FT-IR spectra (3750–1250 cm^–1^) under the same conditions. After introducing humid 400 ppm CO_2_ into the DRIFTS cell, a dramatic peak increase was observed at 3750 cm^–1^, which is attributed to H_2_O adsorption.^74,90,91^ Much faster H_2_O uptake was observed at 25 °C, while the uptake kinetics at −20 °C were extremely slow, as discussed in Figure S7. *In situ* FT-IR desorption experiments were also conducted after humid 400 ppm CO_2_ adsorption at 25 °C to investigate the thermal decomposition behavior of carbamic acid and ammonium carbamate ion pairs formed under humid environments (Figure S20).

The CO_2_/H_2_O TPD and *in situ* FT-IR results show that the formation of carbamic acid and ammonium carbamate ion pairs on MIL-101(Cr)_TEPA(30) and Al_2_O_3__TEPA(20), respectively, was enhanced upon humid 400 ppm CO_2_ adsorption. The enhancement in CO_2_ adsorption under humid conditions became particularly more significant at −20 °C, indicating that the effect of humidity on the DAC performance becomes more significant with decreasing adsorption temperature. The enhanced carbamic acid formation under humid conditions may be because more water-stabilized carbamic acid was formed (via hydrogen bonding) with adsorbed water molecules.^74^ More ammonium carbamate ion pairs also can be formed with water vapor adsorption due to water’s ability to free amine sites for amine–amine or amine-support interactions.^17,80,92,93^ More detailed discussion on the effect of moisture is described in Supporting Information.

Interestingly, a very small peak for the symmetric νCOO^–^ vibration of bicarbonate anions was observed at 1358 cm^–1^ during humid 400 ppm CO_2_ adsorption with the Al_2_O_3__TEPA(20) at 25 and −20 °C, while clear evidence for the formation of bicarbonate under humid conditions was not observed from the MIL-101(Cr)_TEPA(30) (Figure S18). This is probably because ammonium bicarbonate is formed from a paired ammonium carbamate precursor.^74,81,94^ As shown in Figures 3 and S18, since the formation of ammonium carbamate ion pairs is the dominant CO_2_ capture mechanism of Al_2_O_3__TEPA(20), the formation of bicarbonate within Al_2_O_3__TEPA(20) may be more favorable under humid conditions when compared to the MIL-101(Cr)_TEPA(30). However, it has been reported that observation of bicarbonate formation on solid-supported amines under humid CO_2_ capture conditions is challenging with *in situ* FT-IR conditions due either to the small amounts of bicarbonate formed or due to extinction coefficients that make resolution difficult.^95,96^ Thus, the formation of bicarbonate under humid conditions should be further studied with ^13^C solid-state NMR in the future.^96−100^

**Figure 5:**
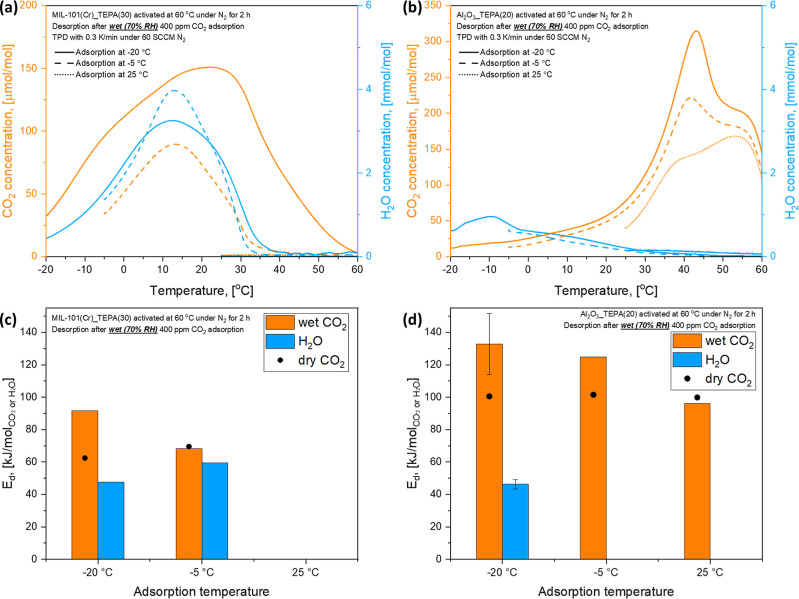
(a/b) CO_2_/H_2_O TPD profiles and (c/d) energy of CO_2_ and H_2_O desorption of (a/c) 30 wt % TEPA-impregnated MIL-101(Cr) and (b/d) 20 wt % TEPA-impregnated γ-Al_2_O_3_ powder adsorbents for humid (70% RH) 400 ppm adsorption at −20, −5, and 25 °C.

The CO_2_/H_2_O TPD profiles of MIL-101(Cr)_TEPA(30) and Al_2_O_3__TEPA(20) are shown in Figure 5a,b, respectively, for different humid CO_2_ adsorption temperature conditions (−20, −5, and 25 °C). The amount of desorbed CO_2_ from the two powder sorbents dramatically increased with decreasing adsorption temperatures from 25 to −20 °C, indicating that the effect of moisture on 400 ppm CO_2_ adsorption became more significant. The binding energy of CO_2_ on the powder sorbents may be affected by H_2_O adsorption. Thus, quantifying the binding energy of CO_2_ and H_2_O under humid conditions is necessary to estimate the energy required for sorbent regeneration. Generally, the isosteric heat of adsorption is calculated from multiple isotherms measured at different temperature conditions. However, measuring the adsorption isotherm of CO_2_ under humid conditions (co-adsorption of CO_2_ and H_2_O) is challenging. Thus, the energies of CO_2_ and H_2_O desorption, *E*_d_, for humid CO_2_ adsorption, were determined based on the TPD method described in Supporting Information. Figures S21 and S22 show the CO_2_/H_2_O TPD profiles of the MIL-101(Cr)_TEPA(30) and Al_2_O_3__TEPA(20), respectively, with varied heating rates for humid CO_2_ adsorption.

The determined *E*_d_s of CO_2_ and H_2_O for humid CO_2_ adsorption are plotted in Figure 5c for MIL-101(Cr)_TEPA(30) and (d) for Al_2_O_3__TEPA(20) along with the results of dry CO_2_ desorption (Figure 4c,d) as a function of adsorption temperature conditions. It should be noted that *T*_m_s for some conditions could not be identified in the TPD profiles due to significant CO_2_ or H_2_O desorption during the N_2_ purging step, and thus, we were not able to determine *E*_d_ for those conditions. The determined desorption energy of water for Al_2_O_3__TEPA(20) was 46.4 kJ/mol_H_2_O_, which is comparable to the heat of vaporization of water in the range of 0–40 °C and 43.4–45.1 kJ/mol_H_2_O_, supporting the condensation-like sorption of water by Al_2_O_3__TEPA(20). A previous study directly measured the heat of adsorption of γ-Al_2_O_3_ via microcalorimetry of water adsorption, and it was −44 kJ/mol_H_2_O_ at a relative water saturation pressure of 0.7 at 25 °C.^71^ In the case of MIL-101(Cr)_TEPA(30), the measured desorption energy of water (47.6–59.4 kJ/mol_H_2_O_) was slightly higher than that of Al_2_O_3__TEPA(20). The reported isosteric heat of water adsorption on MIL-101(Cr) is from −53 to −44 kJ/mol_H_2_O_.^70^ The higher heat of water desorption on the MIL-101(Cr)_TEPA(30) than Al_2_O_3__TEPA(20) might be linked to the smaller pore size of the MOF, as shown in Table 1. Thus, the MIL-101(Cr)_TEPA(30) showed much higher H_2_O uptake than Al_2_O_3__TEPA(20) under all adsorption temperature conditions (Figure 2a).

The calculated energy required for desorption of dry and humid CO_2_, shown in Figure 5c,d, indicates that the binding energy of CO_2_ increases under humid conditions, and the effect of moisture on CO_2_ adsorption is more significant with decreasing adsorption temperature. The difference between wet and dry CO_2_ desorption energies increased with decreasing adsorption temperatures from 25 to −20 °C. At −20 °C, the desorption energy of CO_2_ for Al_2_O_3__TEPA(20) increased from 100.5 kJ/mol_CO_2__ (dry) to 121.1 kJ/mol_CO_2__ under humid conditions. The MIL-101(Cr)_TEPA(30) also showed similar behavior. It can be inferred from the CO_2_ TPD profiles in Figure S17e,f that the increased CO_2_ desorption energy for humid CO_2_ adsorption could be mostly attributed to enhanced strong chemisorption (desorption above 25 °C), which is related to the formation of carbamate species. The ammonium carbamate ion pairs formed under humid conditions may have higher binding energies than the carbamate species formed under dry conditions.^101,102^ Cyclic diamine 2-(aminomethyl)piperidine (2-ampd) functionalized Mg_2_(dobpdc) MOFs also show similar trends. While the DFT-calculated heat of CO_2_ adsorption was −70 kJ/mol for the formation of ammonium carbamate chains under dry conditions, the calculated humid CO_2_ adsorption energy was −88 kJ/mol in the case of the co-adsorption of 1 CO_2_ and 1 H_2_O per diamine, indicating that H_2_O enhances the CO_2_ binding energy for the ammonium carbamate chain formation.^103^

The results in Figure 5c,d initially suggest that much higher desorption energies could be required for operation of DAC process under humid sub-ambient conditions (e.g., −20 °C) than ambient conditions (e.g., 25 °C) due to the significant increase in the binding energy of chemisorbed CO_2_ under humid conditions at −20 °C. However, it should be noted that all the data reported in this study are pseudo-equilibrium values. As discussed in Figure S7, the two powder sorbents have extremely slow H_2_O uptake kinetics when compared to CO_2_ uptake kinetics, especially at sub-ambient temperature conditions (e.g., −20 °C), due to an extremely low absolute humidity level and the effect of cold temperatures on kinetics of chemical processes. Thus, in realistic sub-ambient DAC operation, the H_2_O adsorption working capacity could be very low, depending on the adsorption time and the effect of adsorbed H_2_O on the 400 ppm CO_2_ adsorption may not be significant due to the kinetically limited H_2_O uptake at sub-ambient temperature conditions (e.g., −20 °C).

To support the abovementioned argument, H_2_O uptake profiles of the MIL-101(Cr)_TEPA(30) and Al_2_O_3__TEPA(20) were obtained by integrating the H_2_O breakthrough curves in Figure S7 and are plotted in Figure S23 as a function of time. The dashed lines in the figures depict H_2_O adsorption working capacity at the time required to reach pseudo-equilibrium states (95% of breakthrough) of humid 400 ppm CO_2_ adsorption during the breakthrough experiments in Figure S3. Figure S23 indicates that if the DAC process is operated until the pseudo-equilibrium states at each adsorption temperature, the H_2_O adsorption working capacity could dramatically decrease with decreasing temperatures due to significant kinetic limitation of H_2_O uptake at colder temperatures. The H_2_O adsorption working capacities of MIL-101(Cr)_TEPA(30) and Al_2_O_3__TEPA(20) were decreased from 27.0 and 13.5 mmol/g at 25 °C to 7.7 and 4.1 mmol/g at −20 °C, respectively. The H_2_O uptakes of both sorbent materials were reduced by 3.3–3.5 times when decreasing the adsorption temperature from 25 to −20 °C, indicating that significant energy saving for water desorption may be achieved under sub-ambient DAC operation.

The CO_2_/H_2_O uptakes and total energies for CO_2_ and H_2_O desorption of the two sorbent materials are compared in Table 2 to estimate the energy requirements for sorbent regeneration. These energies are broken down into the energy for desorption (Δ*H*_ads_ + EA_des_) and the sensible heat for heating the sample from the adsorption to the desorption temperature. The energies of CO_2_ and H_2_O desorption were calculated based on the measured CO_2_ and H_2_O uptakes (Figure 2) and the *E*_d_ for CO_2_/H_2_O desorption determined by the TPD method (Figures 4 and 5). Under dry conditions, the MIL-101(Cr)_TEPA(30) sorbents are better sorbent materials for sub-ambient temperature DAC operation (e.g., −20 °C) due to their higher CO_2_ uptakes and lower regeneration energies than Al_2_O_3__TEPA(20). For dry ambient temperature operations (e.g., 25 °C), the Al_2_O_3__TEPA(20) appears to be an advantaged sorbent because of its higher CO_2_ uptake under such conditions. When the sorbent materials are fully saturated with water, their DAC performance is significantly enhanced. However, the sorbent regeneration energy requirement dramatically increases at the same time due to significant amounts of H_2_O adsorption. In particular, DAC operation with MIL-101(Cr)_TEPA(30) is very energy-intensive if water is completely removed in every cycle because of its high H_2_O uptake. However, as discussed above, the sorbent materials are never fully saturated with H_2_O, especially at sub-ambient temperature conditions (e.g., −20 °C) due to kinetically limited H_2_O adsorption. When the estimated H_2_O working capacity (Figure S23) is considered under kinetic conditions in the calculation, both sorbent materials show much lower regeneration energy requirements for DAC operation at −20 than at 25 °C, indicating the advantage of sub-ambient DAC operation.

**Table 2:** DAC Performance and Energies for CO_2_/H_2_O Desorption of MIL-101(Cr)_TEPA(30) and Al_2_O_3__TEPA(20) Sorbent Materials (Desorption at 60 °C Under N_2_)

	MIL-101(Cr)_TEPA(30)		Al_2_O_3__TEPA(20)	
adsorption temperature, [°C]	–20	25	–20	25
Dry				
*q*_CO_2__, [mol/kg]	1.11	0.11	0.81	1.10
*q*_H_2_O_, [mol/kg]	0.00	0.00	0.00	0.00
, [kJ/mol]	62.4	62.4	100.5	99.8
, [kJ/mol]	53.5		46.4	
energy for CO_2_ desorption, [GJ/ton CO_2_]	1.42	1.42	2.28	2.27
energy for H_2_O desorption, [GJ/ton CO_2_]	0.00	0.00	0.00	0.00
sensible heat for sorbent, [GJ/ton CO_2_]	1.46	6.45	1.92	0.62
Kinetically Limited H_2_O Adsorption				
*q*_CO_2__, [mol/kg]	1.87	0.38	1.98	1.33
*q*_H_2_O_, [mol/kg]	7.70	27.00	4.10	13.46
, [kJ/mol]	91.7	62.4	121.1	104
, [kJ/mol]	53.5		46.4	
energy for CO_2_ desorption, [GJ/ton CO_2_]	2.08	1.42	2.75	2.36
energy for H_2_O desorption, [GJ/ton CO_2_]	5.01	86.4	2.18	10.7
sensible heat for sorbent, [GJ/ton CO_2_]	0.87	1.87	0.78	0.51
Full H_2_O Adsorption				
*q*_CO_2__, [mol/kg]	1.87	0.38	1.98	1.33
*q*_H_2_O_, [mol/kg]	28.7	32.7	8.3	13.5
, [kJ/mol]	91.7	62.4	121.1	104
, [kJ/mol]	53.5		46.4	
energy for CO_2_ desorption, [GJ/ton CO_2_]	2.08	1.42	2.75	2.36
energy for H_2_O desorption, [GJ/ton CO_2_]	18.7	104.6	4.44	10.7
sensible heat for sorbent, [GJ/ton CO_2_]	0.87	1.87	0.78	0.51

For DAC operations, Table 2 indicates that the Al_2_O_3__TEPA(20) will be better sorbents than MIL-101(Cr)_TEPA(30) due to its lower H_2_O uptake. However, it should be noted that the MIL-101(Cr) has a weak chemisorption dominant mechanism, and thus, a small temperature swing can be applied. The DAC performance and energies of CO_2_/H_2_O desorption for small temperature swing operation (adsorption at −20 °C and regeneration at 25 °C) are summarized in Table S2. Due to the weak chemisorption dominant mechanism, MIL-101(Cr)_TEPA(30) shows a much higher CO_2_ working capacity, 1.55 mmol/g, than Al_2_O_3__TEPA(20), 0.43 mmol/g, for the small temperature swing operation, resulting in lower sorbent regeneration energy.

Experimental results of this study demonstrate that adjusting interactions between amine and the surface of the support materials can control the CO_2_ capture mechanisms of amine-impregnated sorbent materials, varying from the formation of carbamic acid (weak chemisorption) to ammonium carbamate (strong chemisorption). DAC performances, including CO_2_ uptake and energy requirement for sorbent regeneration, of these two different mechanisms were strongly affected by adsorption temperatures and humidity. These condition-dependent results demonstrate the complexity of designing and operating a DAC process, as there will likely be no optimal sorbent under all conditions in most locations (day/night; summer/winter; rainy/dry conditions), and a material that operates with acceptable performance under all or most of the conditions likely to be encountered is required.^50^

## Conclusions

TEPA (tetraethylenepentamine) was physically impregnated into two mesoporous solid support materials, MIL-101(Cr) and γ-Al_2_O_3_, to explore the effect of support materials on CO_2_ adsorption mechanisms of a model-impregnated amine. These supports have different BET surface area to pore volume ratios and surface chemistry, and the DAC behavior of the amine-impregnated powder sorbents was investigated under a wide range of temperatures (−20 to 25 °C) and humidities (0–70% RH) conditions.

Analysis of 400 ppm CO_2_ and H_2_O adsorption/desorption data on comparably filled MIL-101(Cr) and γ-Al_2_O_3_ provided a means to infer if impregnated amines behave differently within different pore structures when capturing CO_2_. The CO_2_/H_2_O TPD and *in situ* FT-IR results revealed that the impregnated TEPA within MIL-101(Cr) captures CO_2_ primarily by forming carbamic acid (via weak chemisorption, 62.4 kJ/mol_CO_2__), while the TEPA within γ-Al_2_O_3_ forms ammonium carbamate ion pairs (via strong chemisorption, 100.5 kJ/mol_CO_2__) under 400 ppm CO_2_ conditions. Since MIL-101(Cr) has a much higher BET surface area to the pore volume ratio of 1.78 nm^–1^ vs γ-Al_2_O_3_ (0.16 nm^–1^), impregnated amines may more strongly interact with MIL-101(Cr) than γ-Al_2_O_3_. Thus, the carbamic acid within the TEPA-impregnated MIL-101(Cr) adsorbents might be stabilized by surface water molecules or hydroxyl groups. In contrast, ammonium carbamate pairs were primarily formed in the TEPA-impregnated γ-Al_2_O_3_ via amine–amine interactions (as opposed to amine-surface interactions, like in the MIL-101(Cr) samples) during the CO_2_ adsorption, probably due to the weaker interaction between impregnated amines and γ-Al_2_O_3_.

Interestingly, we found that the formation of carbamic acid in the MIL-101(Cr)_TEPA(30) is enhanced at cold temperatures, showing better DAC performance at sub-ambient (e.g., −20 and −5 °C) over ambient (e.g., 25 °C) conditions. Conversely, the formation of ammonium carbamate (a strongly exothermic reaction) on the Al_2_O_3__TEPA(20) is not significantly affected by the adsorption temperature. We speculate that the different trends are because amine mobility dramatically decreases with decreasing temperature conditions. Although the formation of both carbamate and carbamic acid is affected by decreased amine mobility at cold temperatures, it appears that the formation of the ammonium carbamate ion pairs is more strongly impacted by the decrease in amine mobility at cold temperatures than the formation of carbamic acid, due to the higher amine to CO_2_ reaction stoichiometry (2 vs 1). Thus, the thermodynamically favorable reaction at lower temperatures may be suppressed by the decreased amine mobility at cold temperatures (kinetically limited) in the alumina-supported case, where carbamate ion pairs are mainly formed.

Under humid conditions, the formation of ammonium carbamate ion pairs on the Al_2_O_3__TEPA(20) and carbamic acid species on the MIL-101(Cr)_TEPA(30) was enhanced when compared to dry conditions. The enhancement in DAC performance under humid conditions was the most distinct at −20 °C, indicating that the effect of humidity on the DAC performance of the two adsorbents becomes more significant with decreasing adsorption temperatures. The difference between wet and dry CO_2_ desorption energies also increased with decreasing adsorption temperatures from 25 to −20 °C. Additionally, it appears that the two adsorbent materials have different H_2_O adsorption behaviors. The MIL-101(Cr)_TEPA(30) showed much higher H_2_O uptake (28.8–35.2 mmol/g) than the Al_2_O_3__TEPA(20) (8.3–13.5 mmol/g) under all temperature conditions, likely due to the much smaller pore size. However, it is expected that the effect of humidity on the 400 ppm CO_2_ adsorption may not be significant, especially at sub-ambient temperature conditions (e.g., −20 °C) due to the kinetically limited H_2_O uptake. The estimated H_2_O adsorption working capacities of two sorbent materials were reduced by 3.3–3.5× via decreasing the adsorption temperature from 25 to −20 °C. These results suggest that significant energy savings associated with H_2_O and CO_2_ desorption energies could be achieved by operating a DAC process at sub-ambient temperatures (e.g., −20 °C).

There are several limitations to our study. First, although the analysis of different amine-impregnated porous support materials clearly showed that the support materials could affect the CO_2_ adsorption mechanism of impregnated amines, more experiments with a variety of support materials will be required to develop an in-depth understanding of the full range of amine-surface effects. Second, while we observed a very small stretch for bicarbonate anions in the FT-IR spectra of the Al_2_O_3__TEPA(20) upon humid 400 ppm CO_2_ adsorption, it is not clear and definitive evidence for the formation of (or absence of) significant bicarbonate under humid conditions. As discussed in this article, observation of bicarbonate formation on solid-supported amines under humid CO_2_ capture conditions is challenging with *in situ* FT-IR. Finally, this study evaluated powder materials, and any scalable, low pressure drop contactor design will need to incorporate such materials into alternate forms, such as fibers, monoliths, or other structures, which may result in deviations in amine-surface interactions from those found in powder studies.^104−107^

While only two different porous support materials were investigated in this study, the results clearly demonstrate that the CO_2_ capture mechanisms of impregnated amines could be controlled to vary from the formation of carbamic acid (weak chemisorption) to ammonium carbamate (strong chemisorption) by adjusting interactions between the amine and the surface of the support materials. The results also show that H_2_O adsorption behavior is strongly affected by the properties of solid supports. Thus, to achieve optimized DAC performance depending on the range of sub-ambient and ambient temperature operations, proper support materials should be considered for amine impregnation.

## Supporting Information Available

Experimental section, characterization data (N_2_ physisorption and pore size distribution), CO_2_ adsorption behavior (dry and humid 400 ppm CO_2_ breakthrough and CO_2_/H_2_O-TPD data) of TEPA-impregnated MIL-101(Cr) and γ-alumina powder sorbents, and *in situ* FT-IR spectra of TEPA-impregnated MIL-101(Cr) and γ-alumina powder sorbents during CO_2_ adsorption (PDF)
