# Supplementary material for: Support Pore Structure and Composition Strongly Influence the Direct Air Capture of CO2 on Supported Amines
Source: J Am Chem Soc. 2023 Mar 27;145(13):7190–204. doi: 10.1021/jacs.2c12707 (PMC10080690; doi:10.1021/jacs.2c12707)
Supplement: Supplementary file 1 — ja2c12707_si_001.pdf [file ja2c12707_si_001.pdf]

Supporting Information

Support Pore Structure & Composition Strongly Influence Direct Air Capture of CO_2 on Supported Amines

Guanhe Rim, Pranjali Priyadarshini, MinGyu Song, Yuxiang Wang, Andrew Bai, Matthew J. Realff, Ryan P. Lively, * *and Christopher W. Jones* *

School of Chemical & Biomolecular Engineering, Georgia Institute of Technology, 311 Ferst Dr., Atlanta, Georgia 30332-0100, United States

Corresponding Authors

* E-mail: cjones@chbe.gatech.edu, ryan.lively@chbe.gatech.edu

EXPERIMENTAL SECTION

Materials

Chromium(III) nitrate nonahydrate $Cr(NO_3)_3{\cdot}9H_2O$ (99%) and terephthalic acid (H_2BDC) were obtained from Acros Chemicals for MIL-101(Cr) synthesis. γ-Al_2O_3 was obtained from Global Thermostat, LLC. Dimethylformamide (DMF) and tetraethylenepentamine (TEPA, technical grade) were purchased from Sigma-Aldrich. Methanol (MeOH, ACS grade) was purchased from BDH Chemicals. It should be noted that the technical grade TEPA used in this study also contains a small fraction of tertiary amines.[1-2]

Material Synthesis

For amine impregnation, the porous support materials, MIL-101(Cr) and γ-Al_2O_3 were activated at 110-150 °C at 20 mTorr for 24 h. About 500 mg of activated materials were then dispersed in 30 mL MeOH by sonication for 30 min to form a homogeneous suspension. At the same time, 10 mL of TEPA/MeOH solution was prepared and stirred for 30 min to ensure complete dissolution. The solution was mixed with the suspension and stirred at ambient temperature for 24 h. After that, the MeOH was removed via rotary evaporation at 50 ºC under 218 mbar. The obtained material was then further dried under about 10 mTorr vacuum at 40 ºC for > 24 h to obtain the TEPA-impregnated MIL-101(Cr) and γ-Al_2O_3 powder sorbents.

CO_2 Breakthrough Experiments under Dry and Humid Conditions with 400 ppm CO_2

The fixed bed is made of a 1/4” Stainless Steel tube (4 mm ID) with a 5 cm length. A coil shape tube (1/8” Stainless Steel tube) is installed upstream of the breakthrough system for pre-cooling/heating of the inlet gas stream. All dry gas stream was controlled by a mass flow controller (Alicat Scientific). Sample activation was conducted at 60 ℃ under dry N_2 flow (40 sccm) for 2 h

using heating tape. The coil tube and packed bed column containing activated powder sorbents were then immersed into the refrigerated liquid bath of a chiller (Julabo CD-600F) with a set temperature of -20, -5, and 25 ^{o}C. The inlet gas stream was then switched to dry or humid (70% RH at -20, -5, and 25 ^{o}C) 400 ppm CO_2/N_2 (40 sccm) through a 4-way valve to initiate CO_2 adsorption.

A wet gas generator (WETSYS/SETARAM) was used to precisely control the humidity of the inlet gas stream for the humid CO_2 breakthrough experiment. Pre-humidification of sorbent materials was conducted with 40 sccm of humid N_2 gas (70% RH at -20, -5, and 25 °C) prior to the humid CO_2 breakthrough experiment until the water concentration of the outlet gas stream reached 95% of the inlet gas stream (pseudo-equilibrium). The amount of H_2O adsorption was determined based on the H_2O breakthrough curve. The CO_2 and H_2O concentration of the outlet gas stream from the fixed bed was continuously recorded every second using an infrared gas analyzer (LI-840/LI-COR) during the breakthrough experiments.

CO_2/H_2O Temperature Programmed Desorption (TPD) Experiments

CO_2/H_2O-temperature programmed desorption (TPD) experiments were carried out right after dry or humid CO_2 breakthrough experiments in the fixed bed system. After the CO_2 concentration of the outlet gas stream from the fixed bed reached 95% of the inlet CO_2 concentration (400 ppm x 0.95 = 380 ppm), defined here as pseudo-equilibrium, the inlet gas flow was switched to pure N_2 (60 sccm) and the fixed bed was purged for 1-1.5 h at the adsorption temperature conditions (-20, -5, and 25 ^{o}C). The chiller temperature was then slowly increased at a constant heating rate (0.2, 0.3, 0.5, and 0.7 ^{o}C/min) to 60 ^{o}C to desorb CO_2 and H_2O from the powder sorbents. During the CO_2/H_2O TPD process, the CO_2 and H_2O concentrations of the outlet gas stream were continuously measured by a CO_2/H_2O sensor from LiCOR to investigate the CO_2

and H_2O desorption behavior under a wide range of temperature conditions (from the adsorption temperature to 60 ^{o}C).

Measurement of Energy for Dry/Wet CO_2 and H_2O Desorption

The energies for desorption of CO_2 (dry and humid) and H_2O from amine impregnated porous solid sorbents were estimated using a TPD method that was developed by Cvetanovic and Amenomiya.[3] With assumptions of 1st order desorption, a homogeneous adsorption surface, and no re-adsorption, Eq. (1) was developed.

$$2\ln(T_m) - ln\beta = \frac{E_d}{RT_m} + ln\frac{E_d}{AR} \qquad (1)$$

where T_m is the temperature of peak maximum in K (the temperature where shows peak position in CO_2/H_2O TPD curve), β is the constant heating rate in K/s, E_d is the energy for desorption in J/mol, A is a pre-exponential factor for desorption, R is a universal gas constant (8.314 J/mol/K). With experimentally determined T_m for different β, the energy for desorption (E_d) can be obtained from the slope of plot $2\ln(T_m) - ln\beta$ as a function of $\frac{1}{T_m}$.

The regeneration energy for a sorbent in temperature swing adsorption will be provided as the sum of the heat of adsorption (ΔH_{ads}), the activation energy for desorption (EA_{des}), which is typically small, and the sensible heat for heating the sorbent (and associated equipment) from the adsorption to the desorption temperature. In this work, E_d above represents the sum of ΔH_{ads} and EA_{des}. With the exception of Table 2 and Table S2, the contributions of sensible heat are neglected.

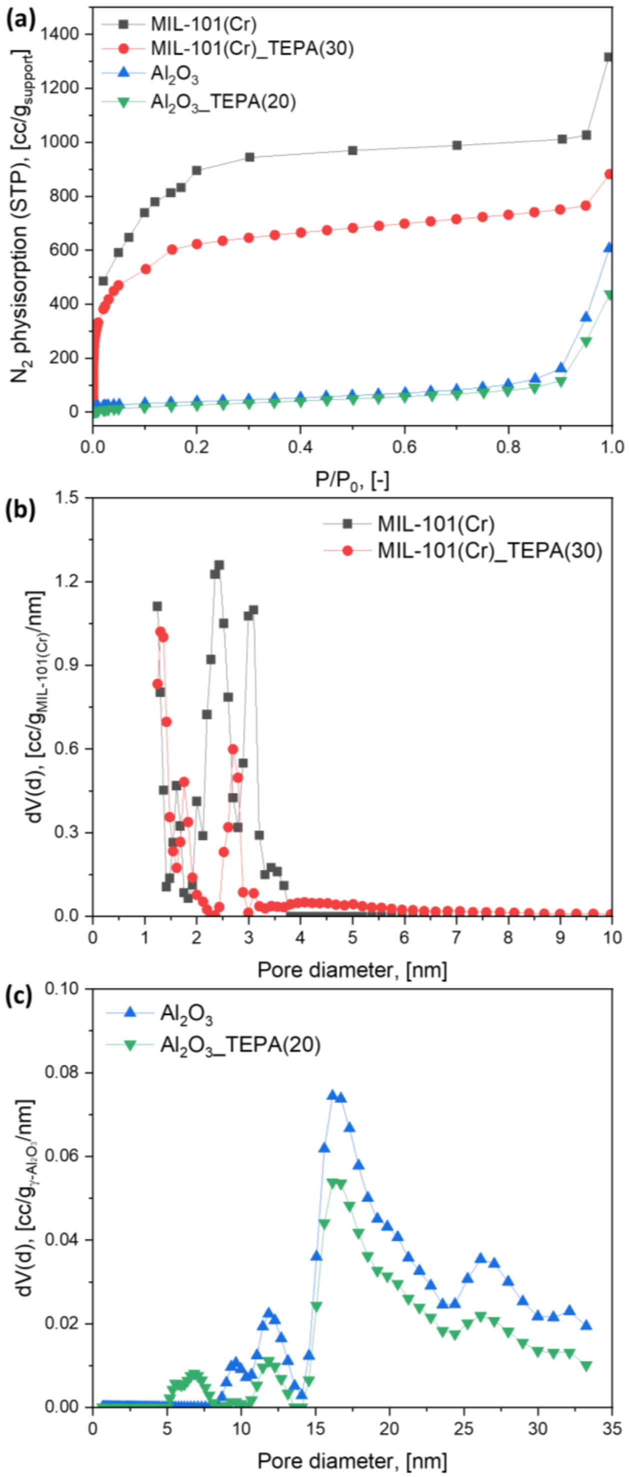

Figure S1. (a) Nitrogen adsorption isotherms of bare and TEPA impregnated MIL-101(Cr) and γ-Al_2O_3. (b) Pore size distribution of bare and 30 wt% TEPA impregnated MIL-101(Cr). (c) Pore size distribution of bare and 20 wt% TEPA impregnated γ-Al_2O_3.

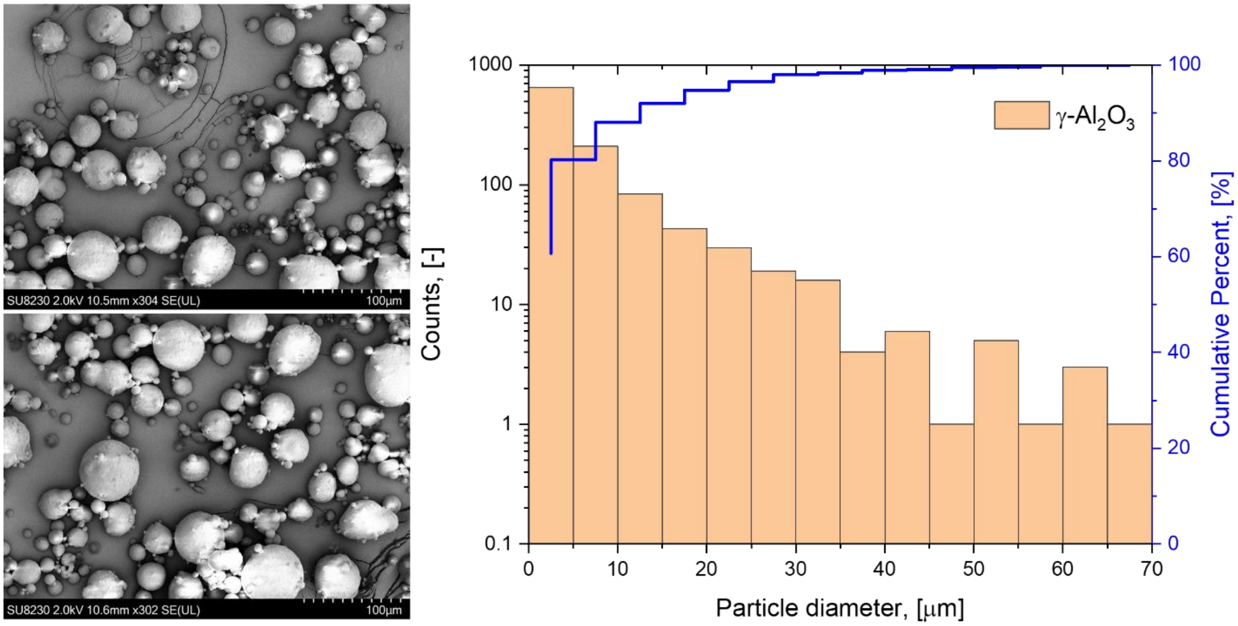

Figure S2. SEM images (left) and measured particle size distribution of γ-Al_2O_3.

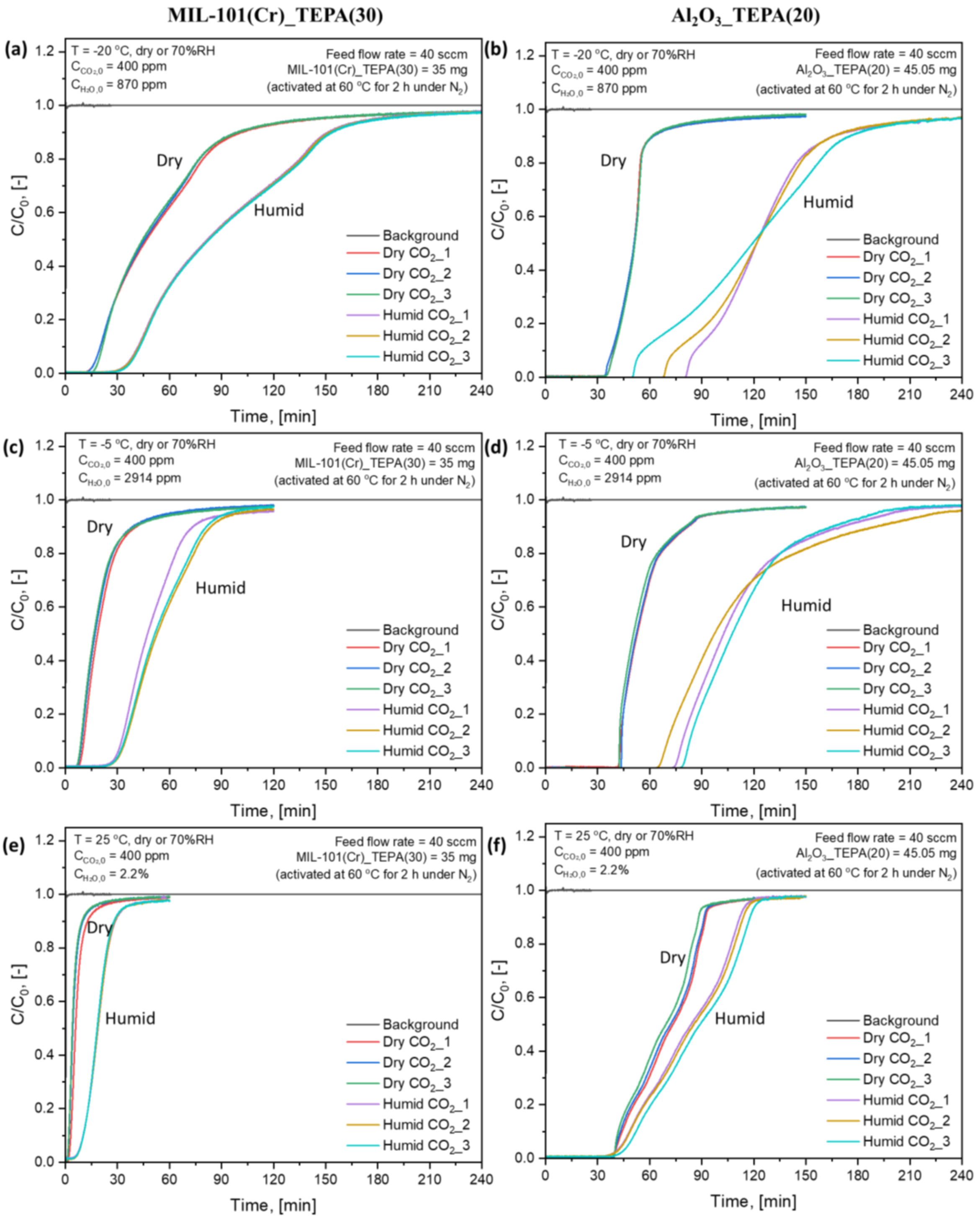

Figure S3. Dry and humid (70%RH) CO_2 breakthrough curves of (a/c/e) 30 wt% TEPA impregnated MIL-101(Cr) and (b/d/f) 20 wt% TEPA impregnated γ-Al_2O_3 powder sorbents at (a/b) -20 ºC, (c/d) -5 ºC, and (e/f) 25 ºC.

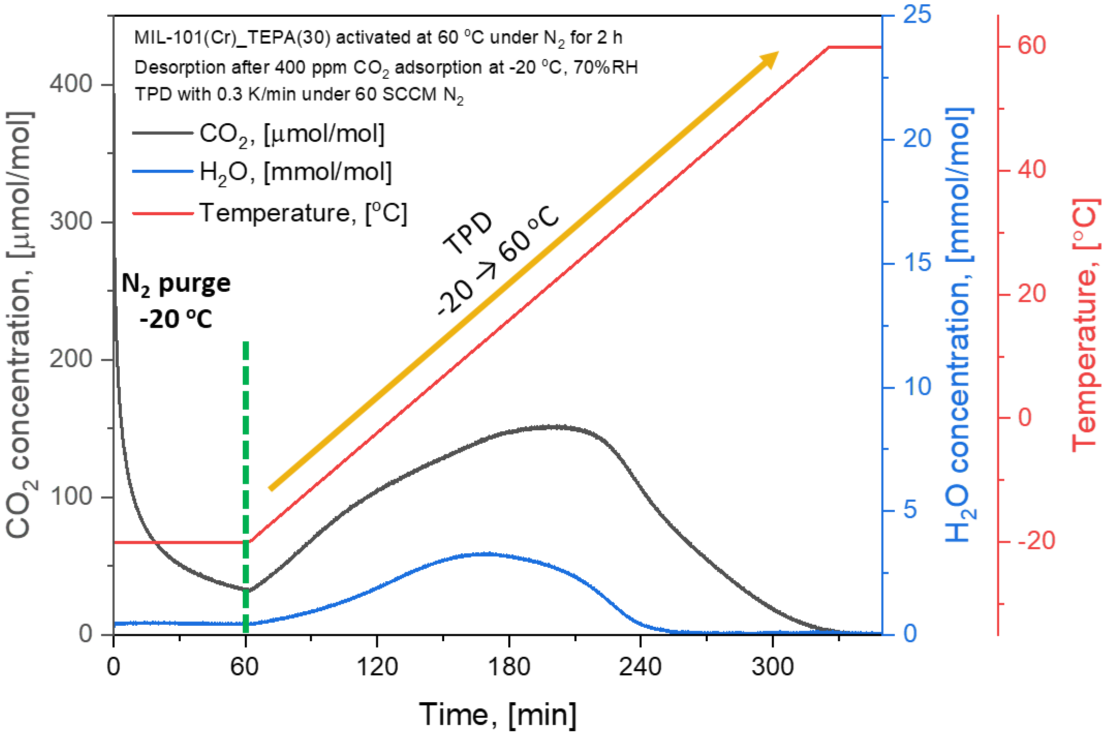

Figure S4. CO_2/H_2O concentration of outlet stream and temperature profiles during CO_2/H_2O TPD experiments with 30 wt% TEPA impregnated MIL-101(Cr). Adsorption conditions: gas, 400 ppm CO_2/N_2; flow rate, 40 sccm; relative humidity, 70%RH; temperature, -20 °C

Figure S4 shows an example of the CO_2/H_2O concentrations of the outlet gas stream and chiller temperature profiles measured during the CO_2/H_2O TPD experiments with 30 wt% TEPA impregnated MIL-101(Cr) on a sample previously subjected to a humid (70% RH) 400 ppm CO_2 breakthrough experiment at -20 °C. Since the area below the CO_2/H_2O concentration profiles in **Figure S4** represents the amount of desorbed CO_2 or H_2O, the desorption of CO_2 and H_2O were quantified by integrating the area under the curve and are plotted in **Figure S5** as a function of time for the dry and humid (70% RH) 400 ppm CO_2 adsorption experiments over MIL-101(Cr)_TEPA(30) and Al_2O_3_TEPA(20) at -20 °C, -5 °C, and 25 °C.

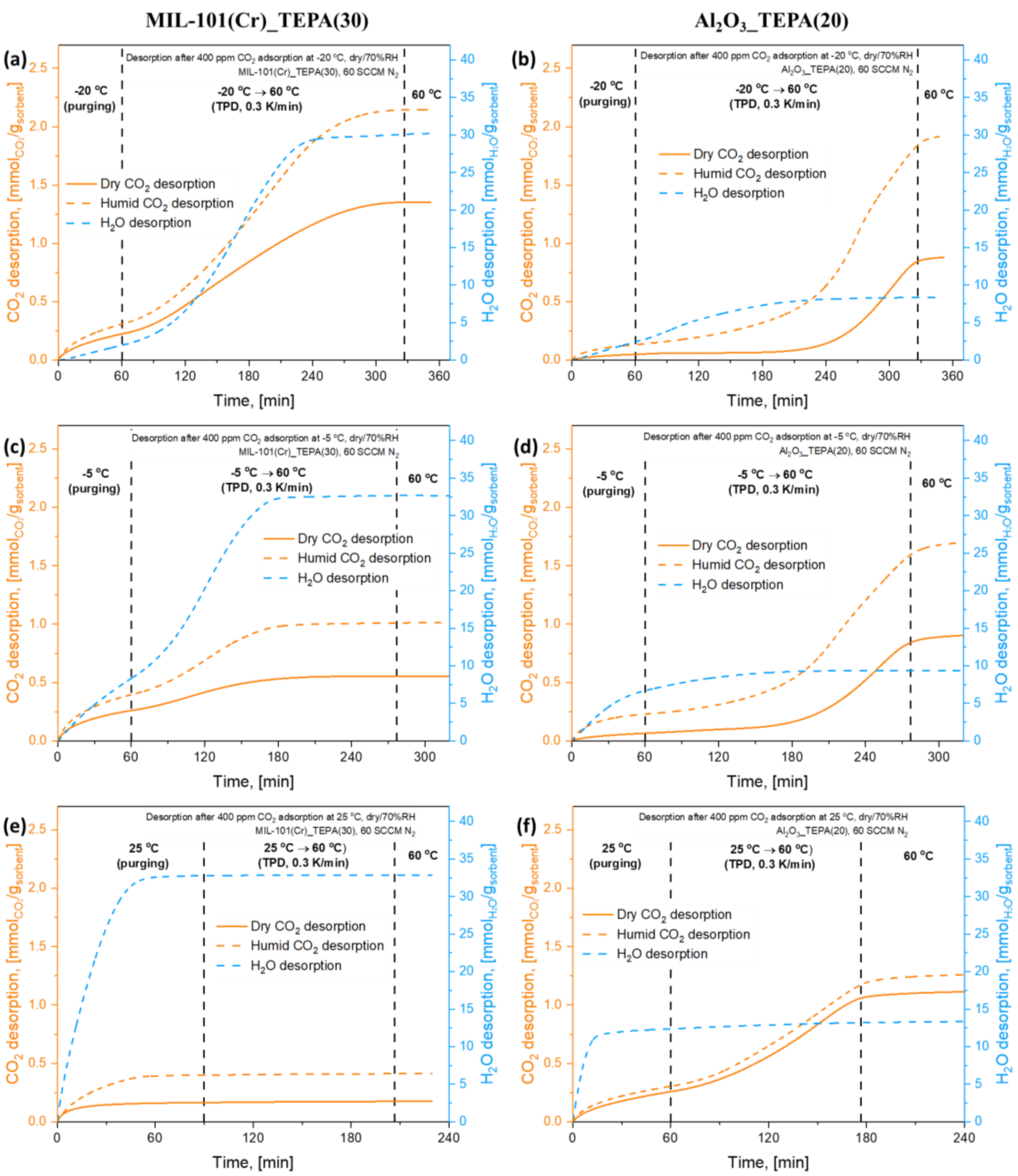

Figure S5. Quantified amount of desorbed CO_2 and H_2O profiles during CO_2/H_2O TPD experiments with (a/c/e) 30 wt% TEPA impregnated MIL-101(Cr) and (b/d/f) 20 wt% TEPA impregnated γ-Al_2O_3 powder sorbents after dry and humid (70%RH) 400 ppm CO_2 adsorption at (a/b) -20 ºC, (c/d) -5 ºC, and (e/f) 25 ºC. Adsorption conditions: gas, 400 ppm CO_2/N_2; flow rate, 40 sccm; activation, 60 ºC under 40 sccm N_2 for 2 - 3 h.

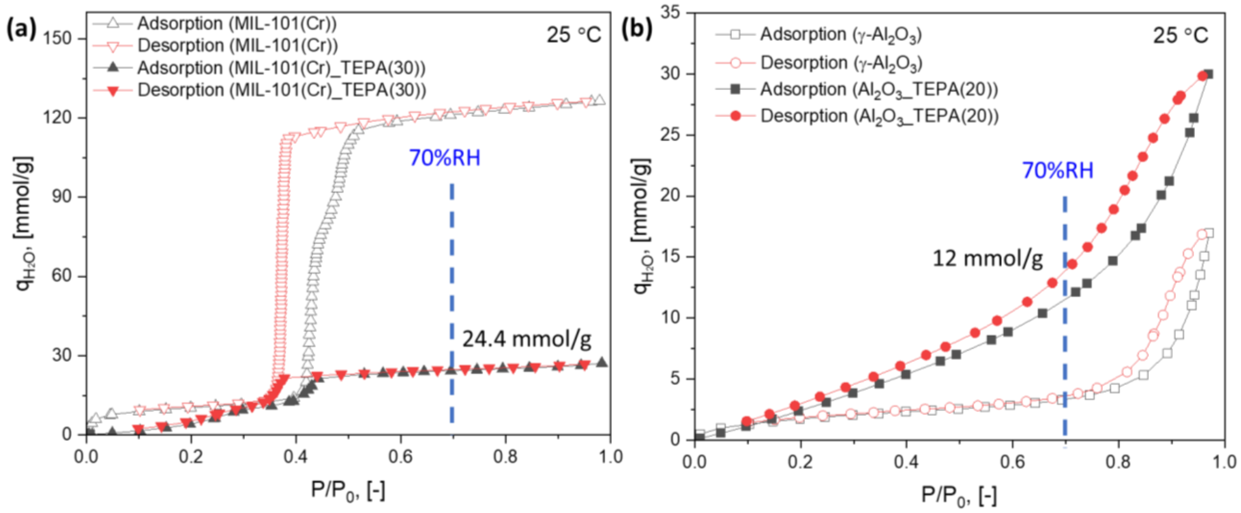

Figure S6. Water vapor adsorption isotherm of (a) MIL-101(Cr)/MIL-101(Cr)_TEPA(30) and (b) γ-Al_2O_3/ Al_2O_3_TEPA(20) at 25 °C. Measured by volumetric vapor sorption analyzer (VSTAR, Anton Paar).

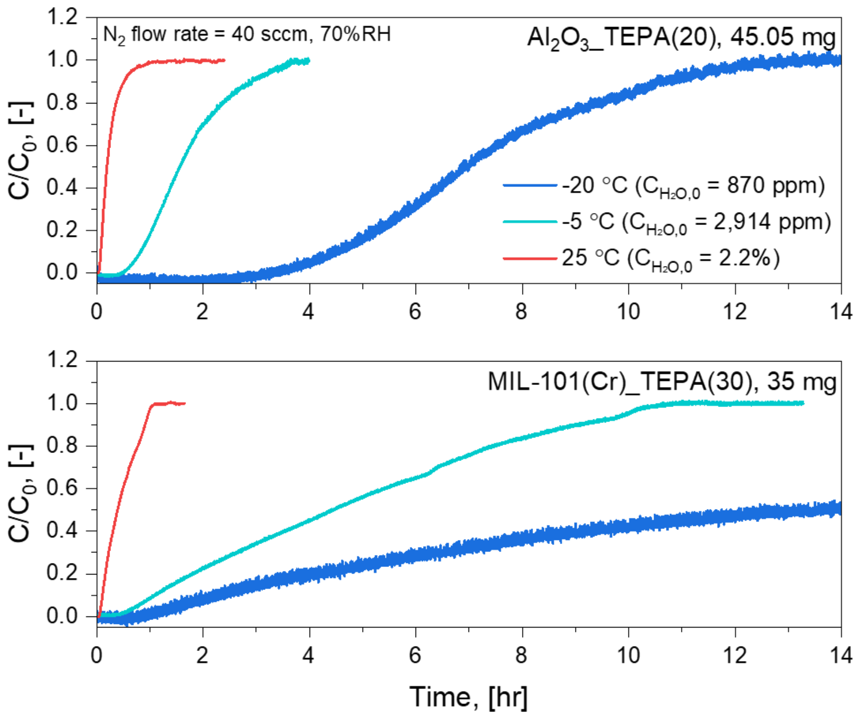

Figure S7. H_2O breakthrough curves of 30 wt% TEPA impregnated MIL-101(Cr) (bottom) and 20 wt% TEPA impregnated γ-Al_2O_3 (top) during pre-humidification. Adsorption conditions: gas, N_2; flow rate, 40 sccm; relative humidity, 70%RH; activation, 60 °C under 40 sccm N_2 for 2 - 3 h.

As shown in the H_2O breakthrough curves measured during the pre-humidification process (**Figure S7**), the time for H_2O saturation of Al_2O_3_TEPA(20) was about 12 h at -20 °C and the full H_2O saturation of MIL-101(Cr)_TEPA(30) was not even achieved within 14 h of exposure during the H_2O breakthrough test, while both sorbents were fully saturated with H_2O within 1 h at 25 °C. Thus, due to the kinetically limited H_2O uptake at sub-ambient temperature conditions (e.g. -20 °C), the H_2O adsorption working capacity of real sub-ambient DAC operation will likely be much lower than the pseudo-equilibrium H_2O uptake shown in **Figure 2(a)**, and it may be even lower than the H_2O adsorption working capacity of ambient temperature DAC operation (e.g. 25 °C), depending on the adsorption time.

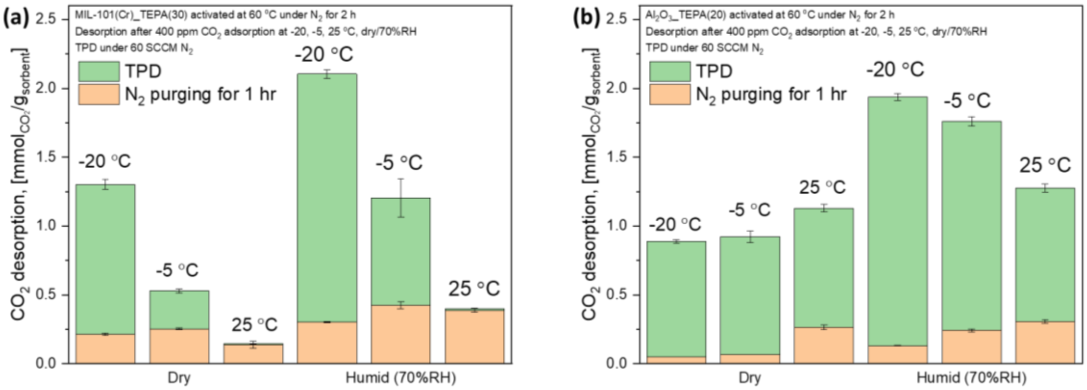

Figure S8. Amount of desorbed dry/humid CO_2 from (a) 30 wt% TEPA impregnated MIL-101(Cr) and (b) 20 wt% TEPA impregnated γ-Al_2O_3 powder adsorbents during the CO_2/H_2O TPD. Adsorption conditions: gas, 400 ppm CO_2/N_2; flow rate, 40 sccm; Relative humidity, 0%RH or 70%RH; activation, 60 ºC under 40 sccm N_2 for 2 - 3 h.

The total amount of desorbed CO_2 is shown in **Figure S8(a)** and **(b)** for MIL-101(Cr)_TEPA(30) and Al_2O_3_TEPA(20), respectively, as a function of adsorption temperature conditions. The amount of CO_2 desorbed was comparable to the pseudo-equilibrium CO_2 uptakes shown in **Figure 1**. The amount of desorbed CO_2 was divided into two sections, (i) CO_2 desorption during the N_2 purging step and (ii) CO_2 desorbed during TPD. During the N_2-purging step at each adsorption temperature, some of the CO_2 was desorbed from the powder sorbents, indicating that physisorption of CO_2 (or weak chemisorption of CO_2) was involved in the adsorption under 400 ppm CO_2 conditions. After the N_2 purging step, a significant amount of chemisorbed CO_2 (or strongly bound CO_2) was desorbed when increasing the temperature up to 60 °C. Interestingly, in the case of dry 400 ppm adsorption at 25 ºC, the MIL-101(Cr)_TEPA(30) sorbent was almost fully regenerated during the N_2 purging step. The amount of desorbed CO_2 increased 2.8 times under humid conditions, indicating that desorbed CO_2 during the N_2 purging step may be dominantly related to weak chemisorption (including physisorption), not physisorption only.

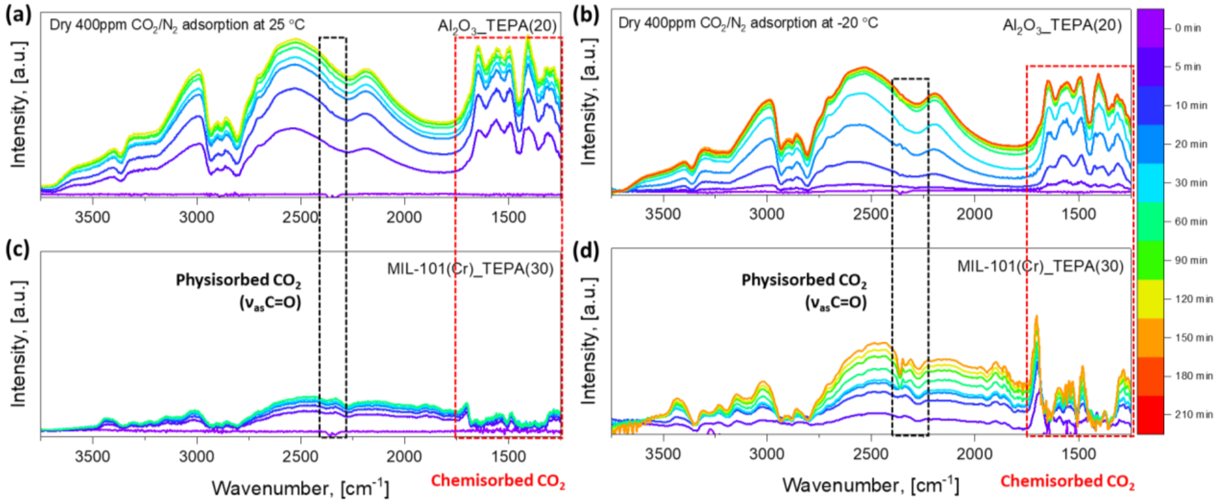

Figure S9. *In situ* FT-IR spectra (3750 cm^{-1} – 1250 cm^{-1}) of (a/b) 20 wt% TEPA impregnated γ-Al_2O_3 and (c/d) 30 wt% TEPA impregnated MIL-101(Cr) powder adsorbents as a function of adsorption time at (a/c) 25 ºC and (b/d) -20 ºC with the activated sample as the background. Adsorption conditions: gas, 400 ppm CO_2/N_2; flow rate, 40 sccm; Relative humidity, dry; activation, 60 ºC under 40 sccm N_2 for 2 - 3 h.

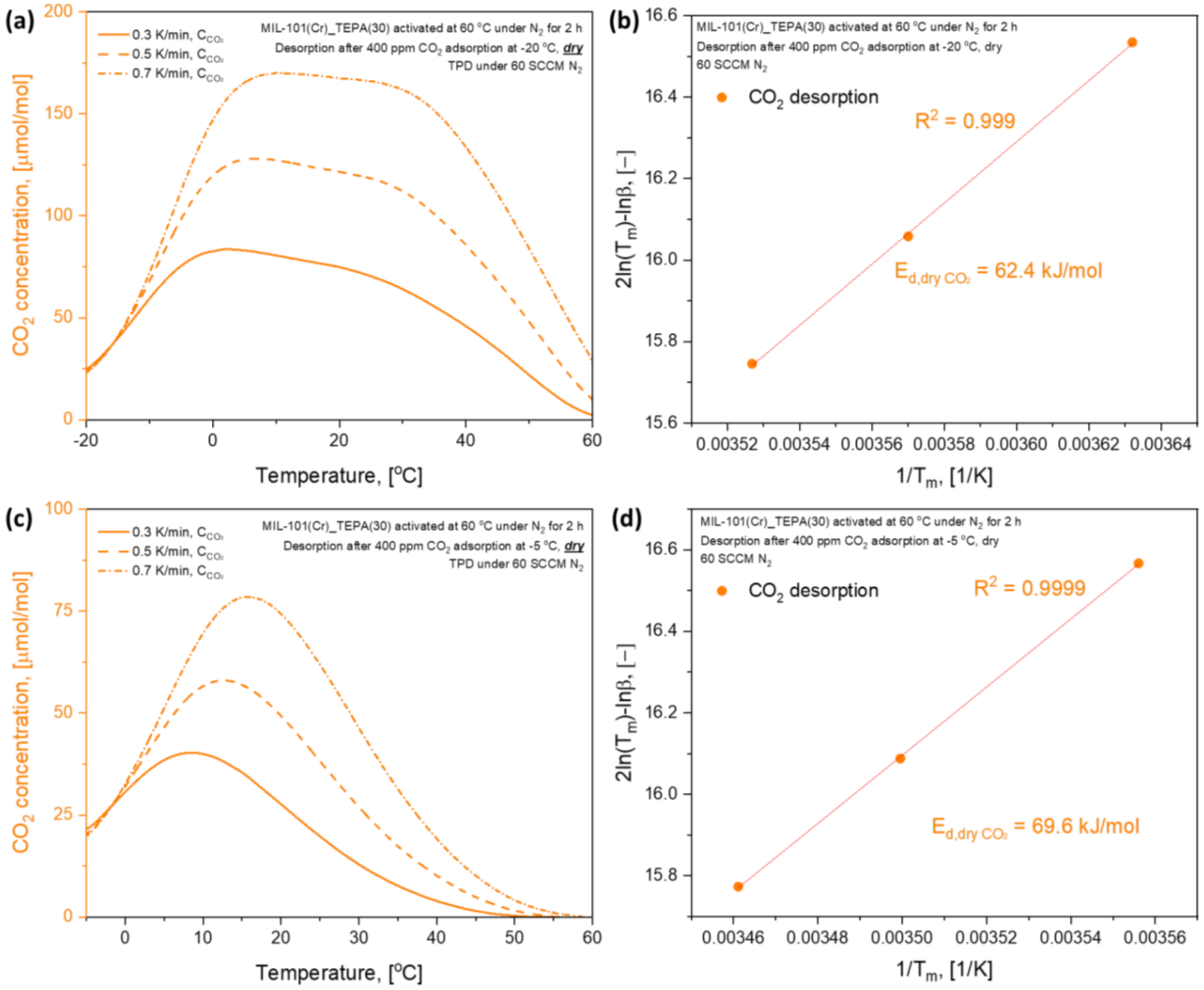

Figure S10. (a/c) CO_2 TPD profiles of 30 wt% TEPA impregnated MIL-101(Cr) powder sorbents with varied heating rate (0.3, 0.5, 0.7 K/min) and (b/d) determination of the energy of CO_2 desorption based on microkinetic analysis for dry 400 ppm CO_2 adsorption at (a/b) -20 ºC and (c/d) -5 ºC. Adsorption conditions: gas, 400 ppm CO_2/N_2; flow rate, 40 sccm.

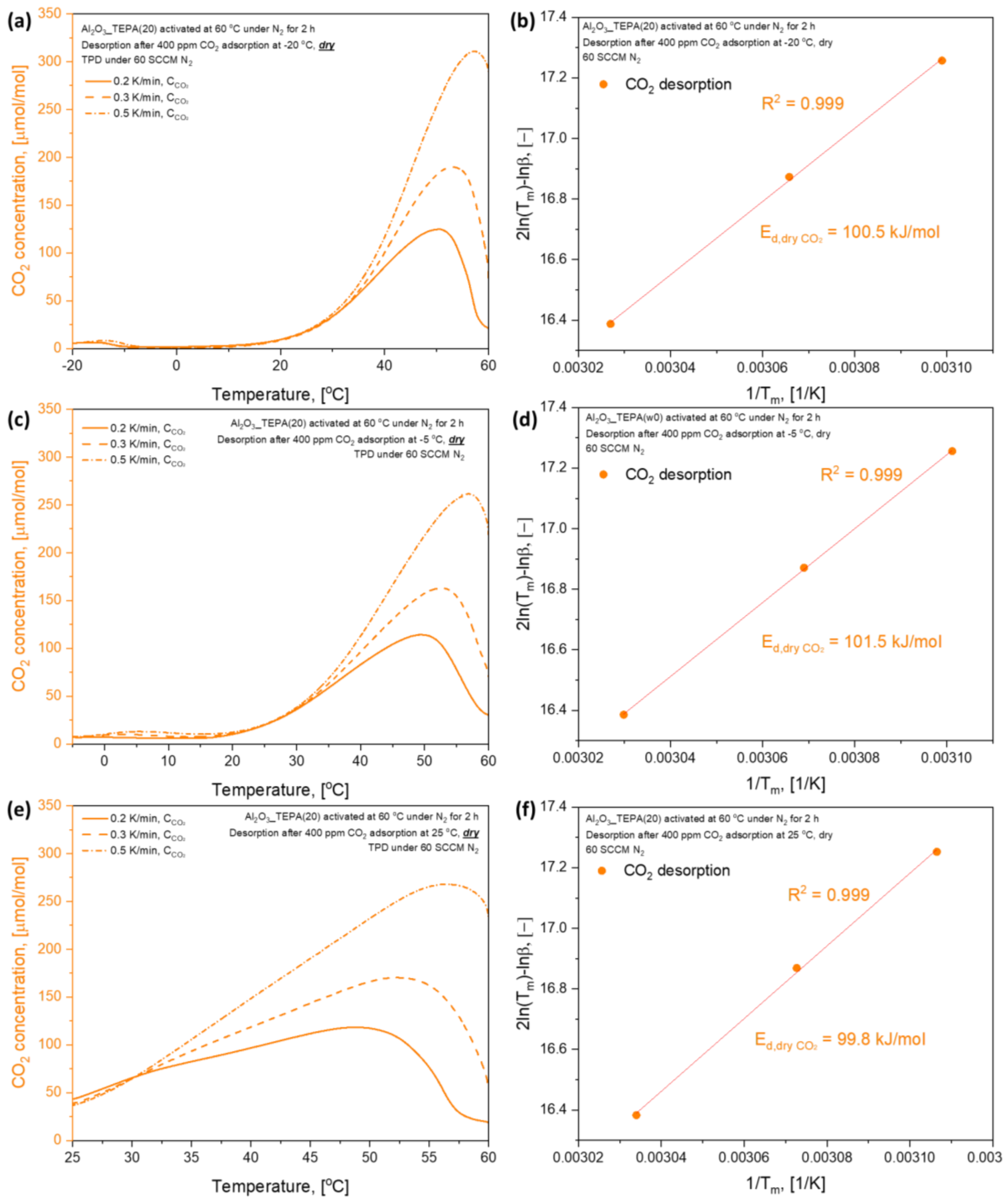

Figure S11. (a/c/e) CO_2 TPD profiles of 20 wt% TEPA impregnated γ-Al_2O_3 powder sorbents with varied heating rate (0.2, 0.3, 0.5 K/min) and (b/d/f) determination of the energy of CO_2 desorption based on microkinetic analysis for dry 400 ppm CO_2 adsorption at (a/b) -20 ºC, (c/d) -5 ºC, and (e/f) 25 ºC. Adsorption conditions: gas, 400 ppm CO_2/N_2; flow rate, 40 sccm.

Panels a and c of **Figure S10** show the dry CO_2 TPD profiles of MIL-101(Cr)_TEPA(30) with varied heating rates (0.3, 0.5, and 0.7 ºC/min) for adsorption temperatures of -20 ºC and -5 ºC, respectively. Since most of captured CO_2 was desorbed during the N_2 purging period at 25 °C (**Figure S5(e)**), it was difficult to obtain a clear CO_2 TPD profile for the 25 °C adsorption temperature. The temperature of the CO_2 desorption peak maximum, T_m, for each heating rate was determined from the profiles. The results clearly show that T_m shifts to higher temperature with increasing heating rates. Eq. (1) was plotted in **Figure S10(b)** and **(d)** for the adsorption temperatures of -20 ºC and -5 ºC, respectively, with the T_m for each heating rate, β, determined in **Figure S10(a)** and **(c)**. The data fitting shows an almost perfect linear relationship and the energy of dry CO_2 desorption from the 30 wt% TEPA impregnated MIL-101(Cr) powder sorbents was directly estimated from the slope. The dry CO_2 desorption energy for the Al_2O_3_TEPA(20) was also determined in the same manner (**Figure S11**).

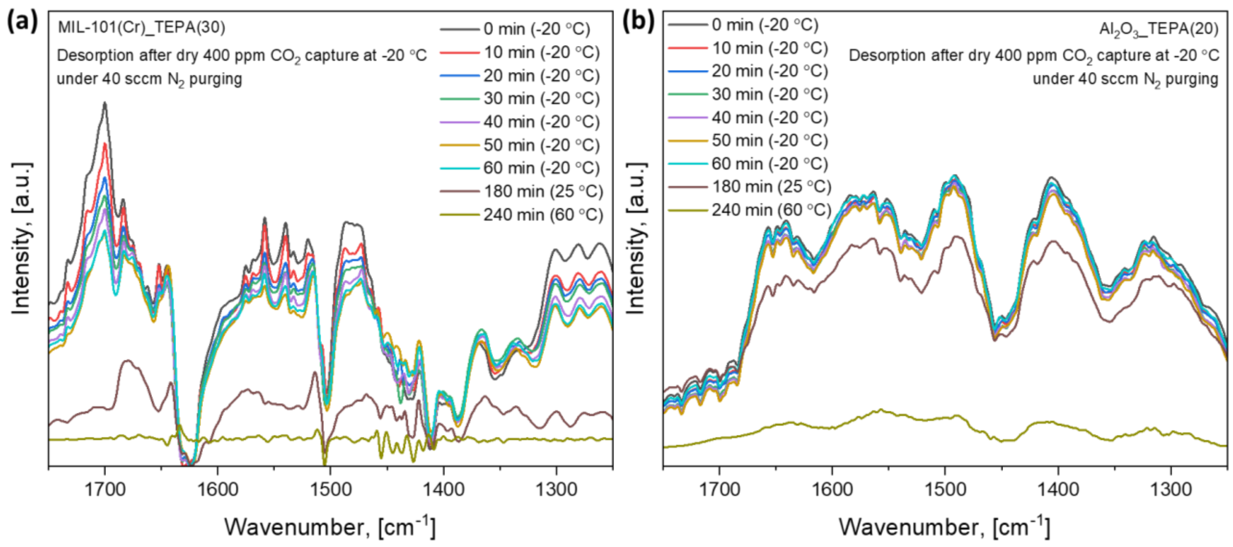

Figure S12. *In situ* FT-IR spectra of (a) 30 wt% TEPA impregnated MIL-101(Cr) and (b) 20 wt% TEPA impregnated γ-Al_2O_3 powder adsorbents as a function of desorption time with the activated sample as the background. Adsorption conditions: gas, 400 ppm CO_2/N_2; flow rate, 40 sccm; relative humidity, dry; adsorption temperature, -20 ºC; activation, 60 ºC under 40 sccm N_2 for 2 - 3 h.

The regeneration process of MIL-101(Cr)_TEPA(30) and Al_2O_3_TEPA(20) saturated with dry 400 ppm CO_2 at -20 °C was monitored with *in situ* FTIR for additional understanding of the thermal decomposition of carbamic acid and carbamate species. First, the 400 ppm CO_2 saturated powder sorbents were purged with N_2 for 1 h at -20 °C, and then the sample was heated to 25 °C and it was held for 2 h. Finally, the temperature of the sorbent was further increased up to 60 °C and it was kept at that condition for 1 h under N_2 flow. During the entire regeneration process, FT-IR spectra of powder sorbents were continuously measured as a function of time. As shown in **Figure S12(a)**, the intensity of the carbamic acid peak (1700 cm^{-1}) in the FT-IR spectra of MIL-101(Cr)_TEPA(30) continuously decreased during the N_2 purging step at -20 °C. The carbamic acid peak was then dramatically reduced at 25 °C, indicating weak chemisorption. On the contrary,

as shown in **Figure S12(b)**, the formed ammonium carbamate ion pairs on the Al_2O_3_TEPA(20) powder sorbents were not decomposed at all during the N_2 purging. The Al_2O_3_TEPA(30) powder sorbents were not effectively regenerated even at 25 °C. At 60 °C, the peak intensity of carbamate species dramatically decreased and almost full decomposition of ammonium carbamate ion pairs occurred, indicating that carbamate species are stronger chemisorption products than carbamic acid.

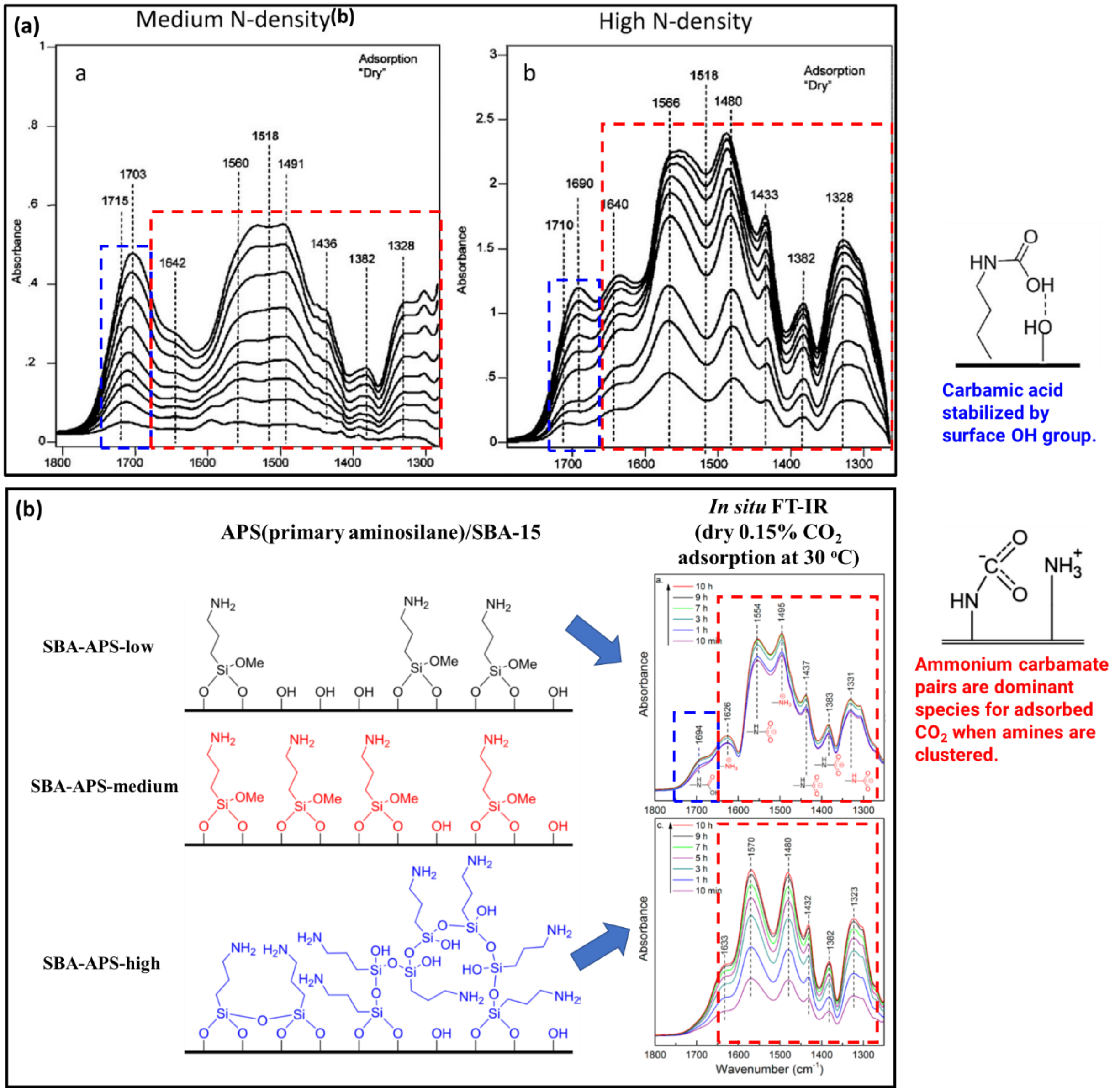

Figure S13. (a) *In situ* FT-IR spectra for a propylamine-modified silica (AMS-6) at medium (left) and high (right) amine densities, reacting with CO_2. Reprinted with permission from [4] Copylight 2011/ACS and [5] Copyright 2019/ELSEVIER. (b) Hypothetical representation of amine materials with low, medium, and high surface coverage and their *in situ* FT-IR spectra as a function of CO_2 adsorption time. Reprinted with permission from [6]. Copyright 2014/ACS.

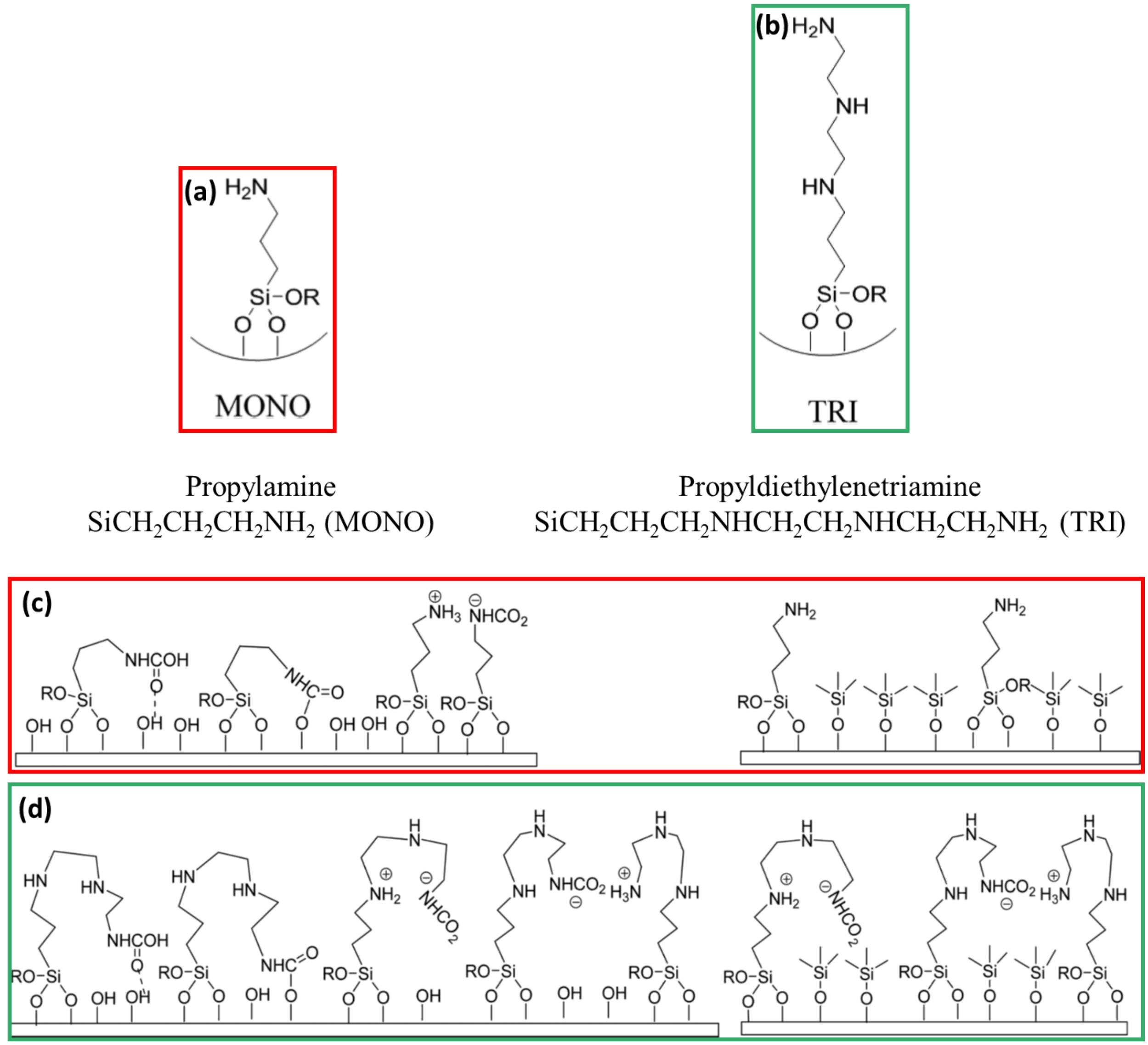

Figure S14. Molecular structure of grafted SBA-15 with amine containing organosilanes; (a) propylamine (MONO) and (b) propyldiethylenetriamine (TRI). Schematic description of hypothesized species resulting from CO_2 chemisorption on organosilane-modified SBA-15 adsorbents; (c) MONO and (d) TRI. Reprinted with permission from [7]. Copyright 2015/ACS.

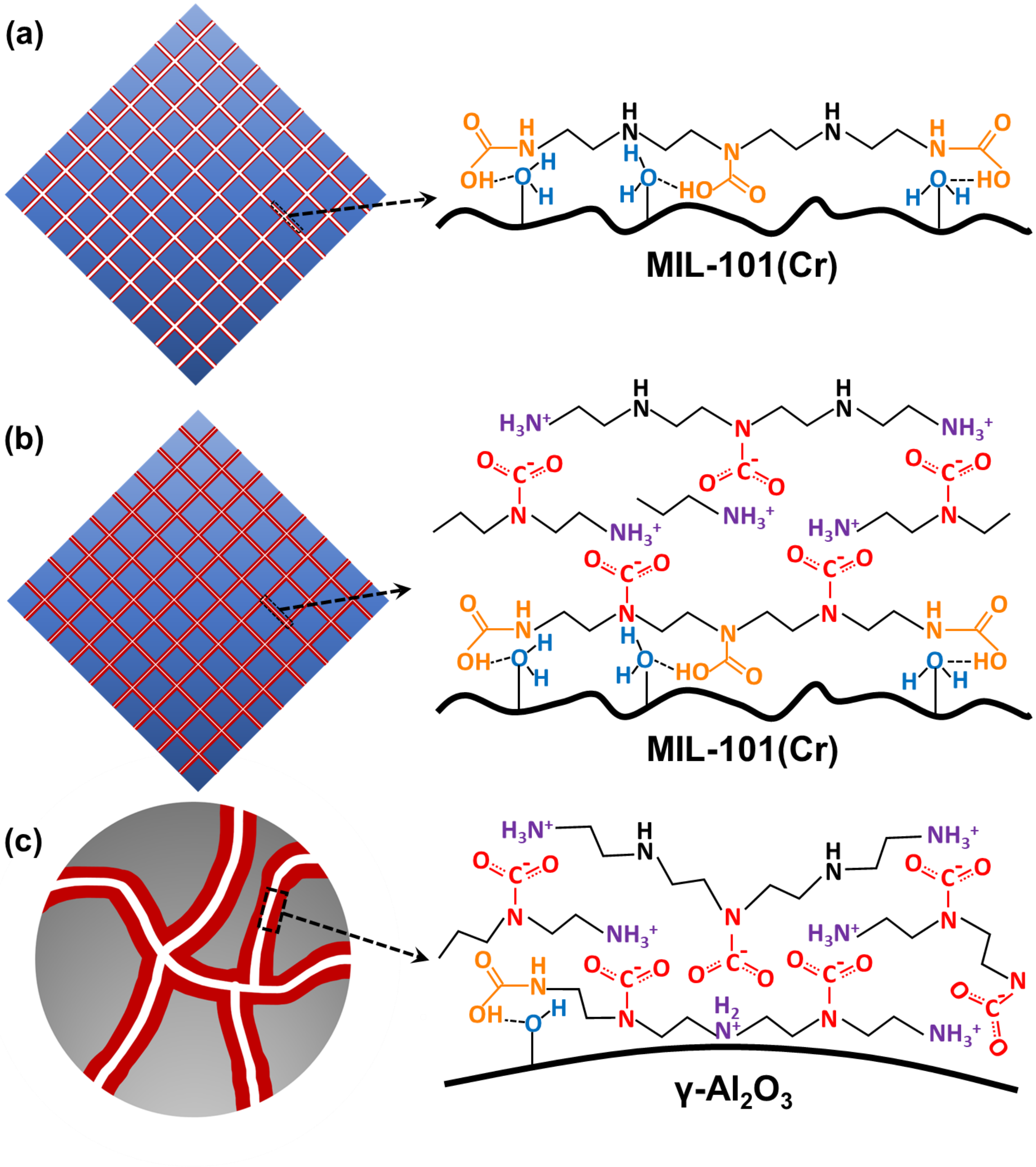

Figure S15. Hypothetical representation of dry CO_2 adsorption mechanism of impregnated TEPA inside MIL-101(Cr) with (a) low and (b) high amine loadings and (c) γ-Al_2O_3 support materials. orange: carbamic acid; red: carbamate ion; purple: ammonium ion; blue: surface water or hydroxyl groups. CO_2/amine ratios are higher than experimental amine efficiencies to minimize the size of the figure.

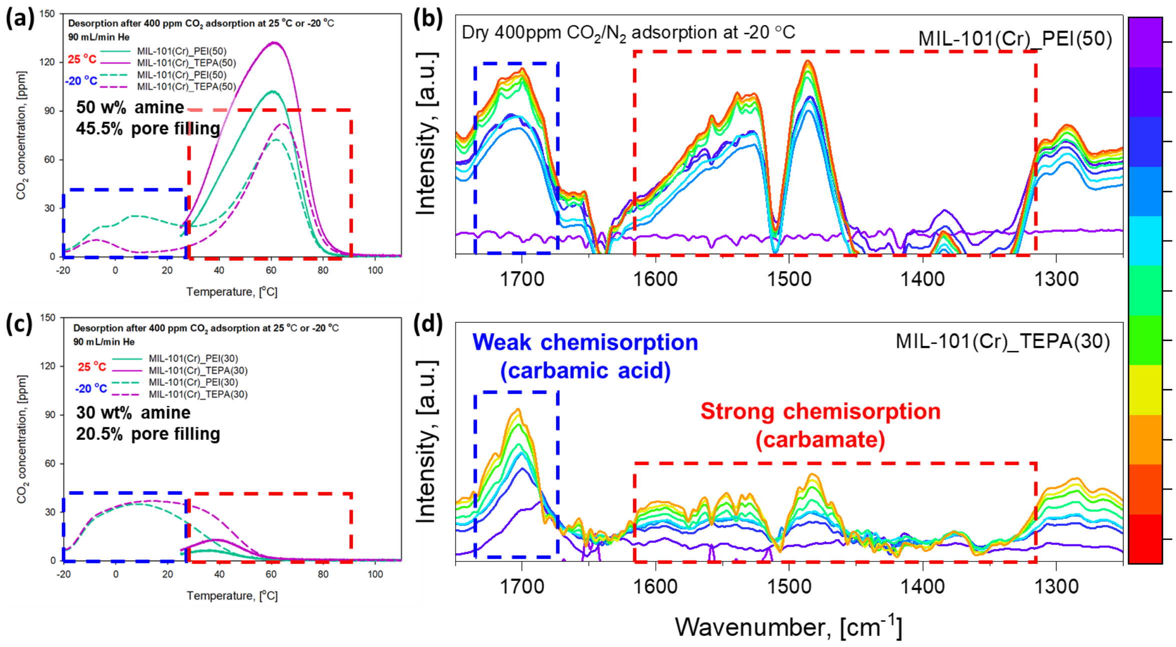

Figure S16. CO_2 TPD profiles of PEI and TEPA impregnated MIL-101(Cr) powder sorbents with (a) 50 wt% and (c) 30 wt% amine loadings. Reprinted with permission.[2] Copyright 2022/ACS. *In situ* FT-IR spectra of (b) 50 wt% PEI and (d) 30 wt% TEPA impregnated MIL-101(Cr) powder adsorbents as a function of adsorption time with the activated sample as the background. Adsorption conditions: gas, 400 ppm CO_2/N_2; flow rate, 40 sccm; Relative humidity, dry; activation, 60 ºC under 40 sccm N_2 for 2 - 3 h.

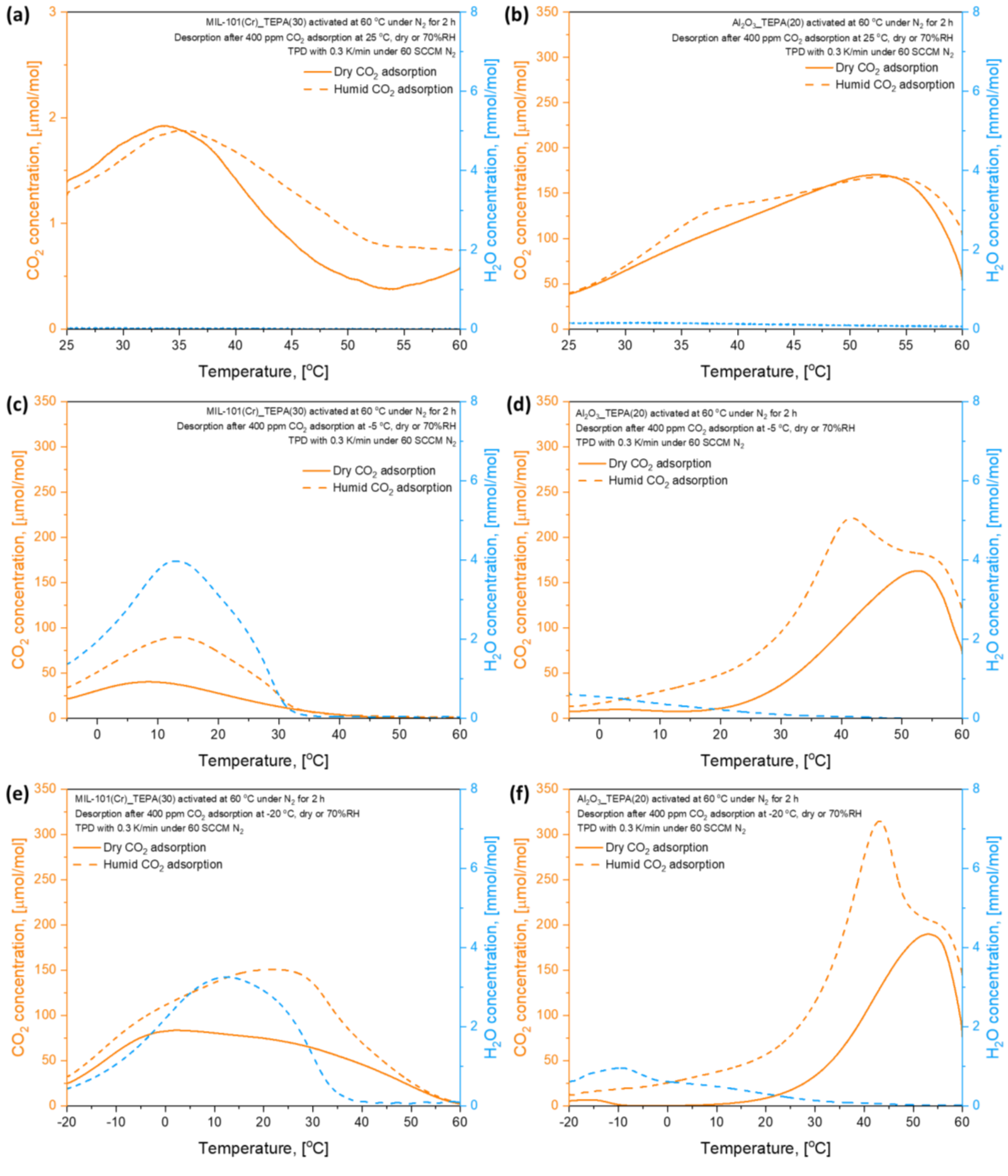

Figure S17. CO_2/H_2O TPD profiles of (a/c/e) 30 wt% TEPA impregnated MIL-101(Cr) and (b/d/f) 20 wt% TEPA impregnated γ-Al_2O_3 powder adsorbents for dry and humid (70%RH) 400 ppm adsorption at (a/b) 25 ºC, (c/d) -5 ºC, and (e/f) -20 ºC.

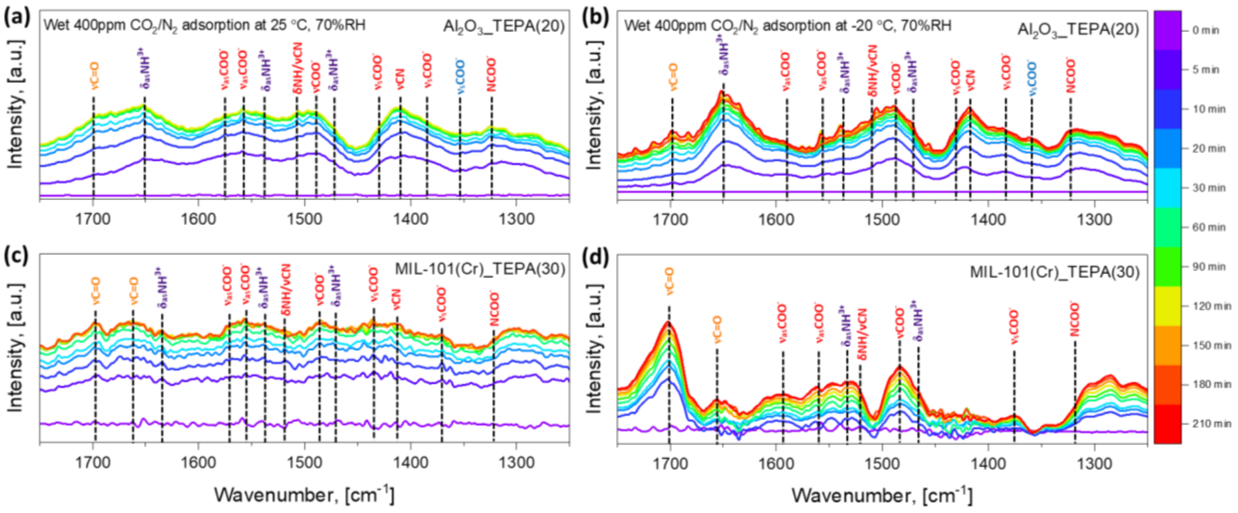

Figure S18. *In situ* FT-IR spectra as a function of humid (70%RH) 400 ppm CO_2/N_2 adsorption time for (a/b) 20 wt% TEPA impregnated γ-Al_2O_3 and (c/d) 30 wt% TEPA impregnated MIL-101(Cr) powder adsorbents at (a/c) 25 ºC and (b/d) -20 ºC with the activated sample as the background. orange: carbamic acid, red: carbamate ion, purple: ammonium ion, blue: bicarbonate.

As shown in **Figure 2(b)**, the MIL-101(Cr)_TEPA(30) adsorbed dry and humid 400 ppm CO_2 via weak chemisorption at 25 °C and the amount of weak chemisorption was enhanced from 0.14 mmol/g (dry) to 0.39 mmol/g under humid conditions. Since the weakly captured CO_2 was almost fully desorbed during the N_2 purging step at 25 °C, meaningful CO_2 TPD profiles of dry and humid 400 ppm CO_2 adsorption could not be obtained. As shown in **Figure S17(a)**, the concentration of desorbed CO_2 was very low (under 2 ppm) throughout the experiment. Likewise, since all the physisorbed H_2O was desorbed during the N_2 purging step (**Figure S5(e)**), the H_2O desorption profile was not observed during the heating from 25 °C to 60 °C.

The *in situ* FT-IR study shown in **Figure S18(c)** identified the adsorbed CO_2 species on the MIL-101(Cr)_TEPA(30) material at 25 °C under humid conditions. The equilibrium intensity

of the overall spectra between 1750 cm^{-1} and 1250 cm^{-1} was significantly enhanced when compared to the dry 400 ppm CO_2 adsorption conditions shown in **Figure 3(c),** indicating that the amount of chemisorbed CO_2 species increased with humidity. Enhanced carbamic acid peaks at 1700 cm^{-1} and 1658 cm^{-1} were observed under humid conditions, which is probably because more water-stabilized carbamic acid was formed (via hydrogen bonding) with adsorbed water molecules.[8] It appears that the formation of ammonium carbamate ion pairs was also enhanced upon humid 400 ppm CO_2 adsorption at 25 °C. It is generally reported that more ammonium carbamate ion pairs are formed with water vapor adsorption when amine-based sorbents capture CO_2 due to water's ability to free amine sites for amine-amine or amine-support interactions.[4, 9-11]

Interestingly, the captured CO_2 as both carbamic acid and carbamate ions under humid conditions was almost fully desorbed during the N_2 purging at 25 °C, indicating weak chemisorption (**Figure 2(b)**). The formed ammonium carbamate ion pairs under humid conditions may have a lower heat of desorption or may be easily decomposed via water desorption without heating. The *in situ* FT-IR spectra collected during the regeneration of the MIL-101(Cr)_TEPA(30) after humid 400 ppm CO_2 adsorption at 25 °C also support this argument. As shown in the *in situ* FT-IR desorption experiments (**Figure S20(a)**), the overall peak intensity of chemisorbed species (carbamic acid and ammonium carbamate ion pair) was dramatically reduced along with significant water desorption during the N_2 purging step and it was then followed by regeneration of a small fraction of strongly chemisorbed species at 60 °C.

The effects of humidity on the DAC performance of Al_2O_3_TEPA(20) at 25 °C were not as significant, as shown in **Figure 2(c)**. Both weak and strong chemisorption were slightly enhanced under humid conditions and the overall 400 ppm CO_2 uptake (including weak and strong chemisorption) increased to 1.28 mmol/g, which is only about 13% higher than that of dry

conditions (1.13 mmol/g). Similar to the MIL-101(Cr)_TEPA(30), the physisorbed water on the surface of Al_2O_3_TEPA(20) at 25 °C was almost fully desorbed by N_2 purging, as shown in (**Figure S5(f)**), resulting in no H_2O desorption profile during the CO_2/H_2O TPD experiment (**Figure S17(b)**). The enhanced strong chemisorption under humid conditions can be observed from the CO_2 TPD profiles. The overall CO_2 desorption curve after humid CO_2 adsorption was slightly increased when compared to that of dry CO_2 adsorption, indicating that more ammonium carbamate ion pairs were formed under humid conditions, as also reported in previous studies.[4, 9-11] One key difference can be observed for humid 400 ppm CO_2 adsorption on the Al_2O_3_TEPA(20) material at 25 °C in **Figure S18(a)**; a new carbamic acid peak at 1700 cm^{-1} appeared under humid conditions, which was not observed for dry CO_2 adsorption, as shown in **Figure 3(a)**. As discussed above, the formation of carbamic acid species upon humid 400 ppm CO_2 adsorption may be because water-stabilized carbamic acid was formed with adsorbed water molecules.[8] *In situ* FT-IR desorption experiments with the Al_2O_3_TEPA(20) (**Figure S20(b)**) show that the formed carbamic acid species (weak chemisorption) under humid conditions were almost fully decomposed along with significant water desorption during the N_2 purging step at 25 °C, while most carbamate species (strong chemisorption) were thermally decomposed at 60 °C.

At -5 °C and -20 °C adsorption temperatures, the enhancement in the CO_2 uptakes of MIL-101(Cr)_TEPA(30) and Al_2O_3_TEPA(20) adsorbents under humid conditions is clearly shown in **Figure 2(b)** and **(c)**. While mostly enhanced weak chemisorption (formation of carbamic acid) was observed from the CO_2 TPD profiles of MIL-101(Cr)_TEPA(30) under humid conditions (**Figure S17(c)**), the enhanced CO_2 uptake of Al_2O_3_TEPA(20) under humid conditions was dominantly attributed to an increase in strong chemisorption (formation of carbamate), as shown in **Figure S17(d)**. This behavior became more distinct for humid 400 ppm adsorption at -20 °C

compared to -5 ºC and 25 ºC, as shown in **Figure S17(e)** and **(f),** indicating that the effect of humidity on the DAC performance of the two adsorbents becomes more significant with decreasing adsorption temperatures. Additionally, it appears that the two adsorbent materials have different H_2O desorption behaviors. The MIL-101(Cr)_TEPA(30) showed a significant H_2O desorption peak centered at 10 °C – 15 °C and almost full H_2O desorption was achieved at 30 °C for the humid CO_2 adsorption at -5 °C and -20 °C (**Figure S17(c)** and **(e)**). A distinct H_2O desorption peak was not observed from the H_2O TPD profile of Al_2O_3_TEPA(20) for the -5 °C adsorption temperature condition (**Figure S17(d)**) because significant water desorption occurred during the N_2 purging step at -5 °C, as shown in **Figure S5(d)**. An H_2O desorption peak centered at -9.7 °C, which is lower than that of MIL-101(Cr)_TEPA(30), was confirmed for humid CO_2 adsorption at -20 °C. This suggests that the Al_2O_3_TEPA(20) has a lower heat of H_2O adsorption (or desorption) than the MIL-101(Cr)_TEPA(30).

To identify the chemisorbed CO_2 species on the TEPA impregnated γ-Al_2O_3 and MIL-101(Cr) sorbents at -20 °C under humid conditions, in situ FT-IR spectroscopy was also conducted under humid 400 ppm CO_2 (70% RH) flow without pre-humidification of the sorbent materials. As shown in **Figure S18(d)**, the overall FT-IR spectra of MIL-101(Cr)_TEPA(30) under humid conditions are similar to the spectra obtained under the dry conditions shown in **Figure 3(d),** indicating that carbamic acid and ammonium carbamate ion pairs were formed under both dry and humid conditions at -20 °C. However, more intense and distinct FT-IR peaks were observed under humid conditions compared to dry conditions. Especially, it appears that enhancement in the intensity of carbamic acid peaks at 1700 cm^{-1} and 1658 cm^{-1} is more significant than that of ammonium carbamate ion pairs, indicating that weak chemisorption (formation of carbamic acid) was dominantly enhanced under humid conditions. This is also confirmed in **Figure S17(e)**. The

measured FT-IR spectra of Al_2O_3_TEPA(20) under humid conditions at -20 °C are shown in **Figure S18(b)** as a function of adsorption time. When compared to the FT-IR spectra under dry conditions at -20 °C (**Figure 3(b)**), sharper, more intense peaks of ammonium carbamate ion pairs were observed under the humid conditions at -20 °C along with a small peak at 1700 cm^{-1}, which is assigned as the νC=O vibration of carbamic acid. This result suggests that strong chemisorption (formation of carbamate species) on the Al_2O_3_TEPA(20) powder sorbents was dominantly enhanced when capturing humid CO_2 at -20 °C with insignificant enhancement in weak chemisorption (formation of carbamic acid), which is consistent with **Figure S17(f)**.

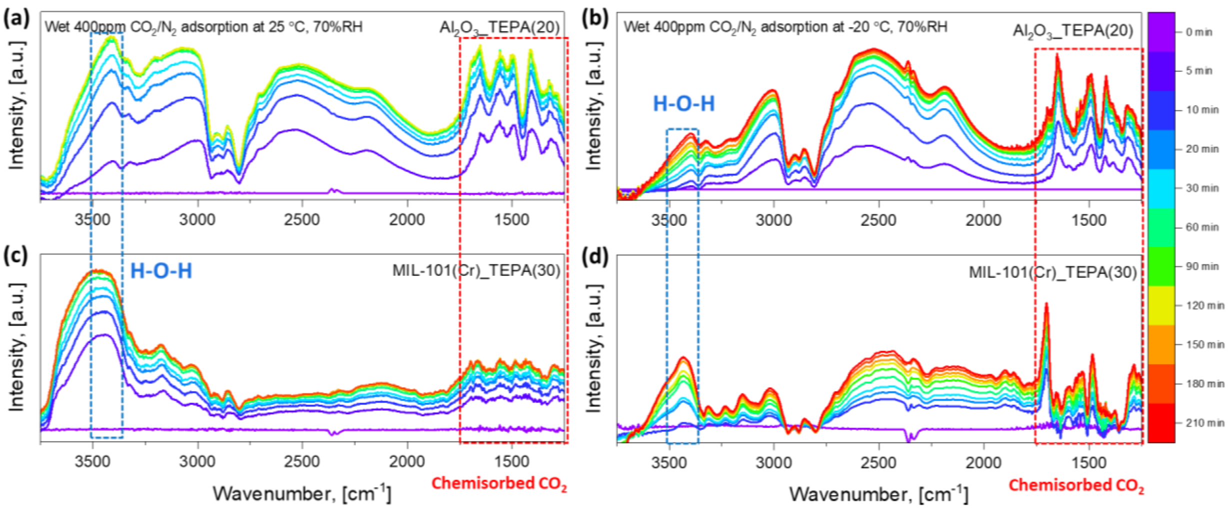

Figure S19. *In situ* FT-IR spectra (3750 cm^{-1} – 1250 cm^{-1}) of (a/b) 20 wt% TEPA impregnated γ-Al_2O_3 and (c/d) 30 wt% TEPA impregnated MIL-101(Cr) powder adsorbents as a function of adsorption time at (a/c) 25 ºC and (b/d) -20 ºC with the activated sample as the background. Adsorption conditions: gas, 400 ppm CO_2/N_2; flow rate, 40 sccm; relative humidity, 70%RH; activation, 60 ºC under 40 sccm N_2 for 2 - 3 h.

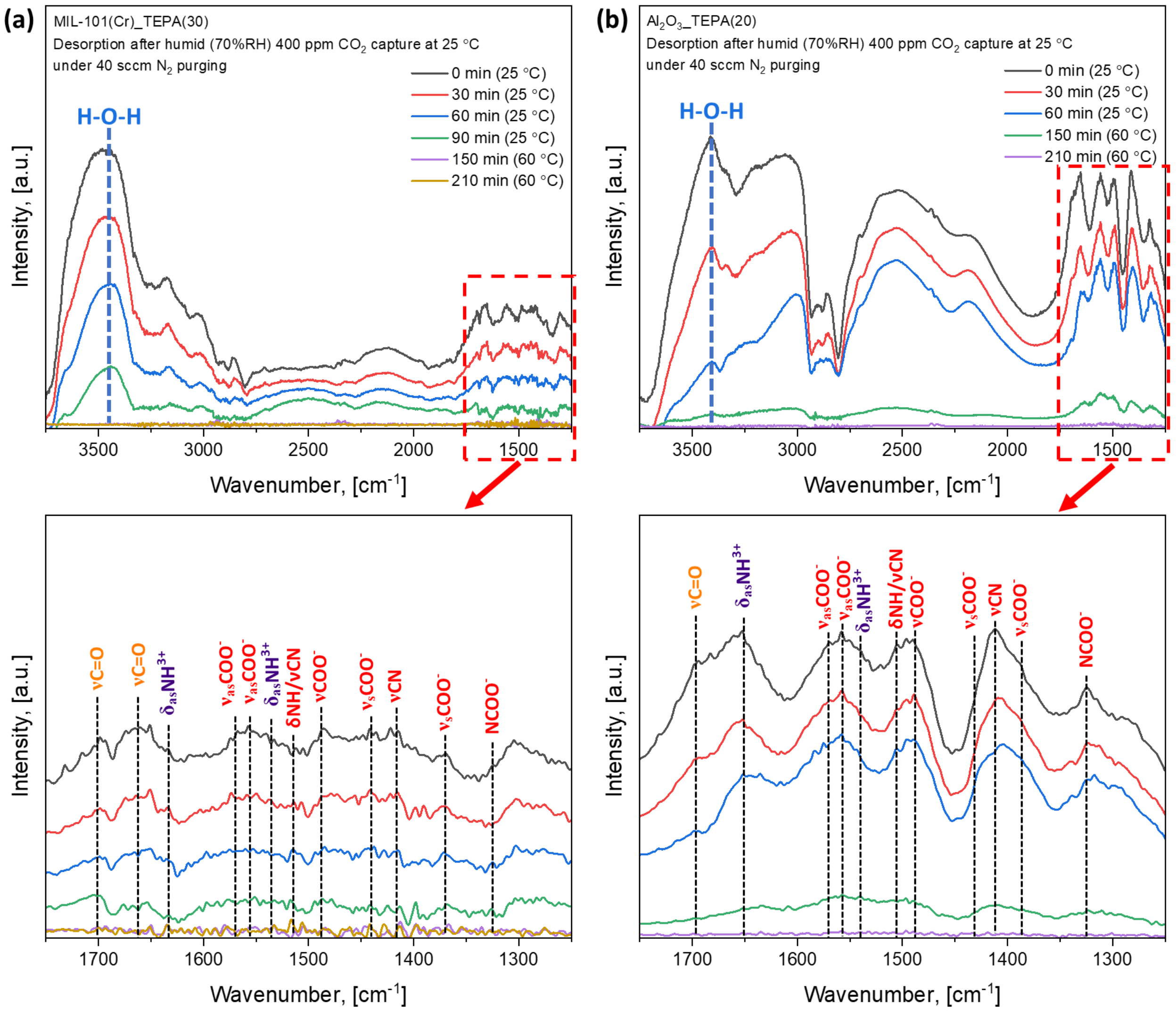

Figure S20. *In situ* FT-IR spectra of (a) 30 wt% TEPA impregnated MIL-101(Cr) and (b) 20 wt% TEPA impregnated γ-Al_2O_3 powder adsorbents as a function of desorption time with the activated sample as the background. Adsorption conditions: gas, 400 ppm CO_2/N_2; flow rate, 40 sccm; relative humidity, 70%RH; adsorption temperature, 25 ºC; activation, 60 ºC under 40 sccm N_2 for 2 - 3 h.

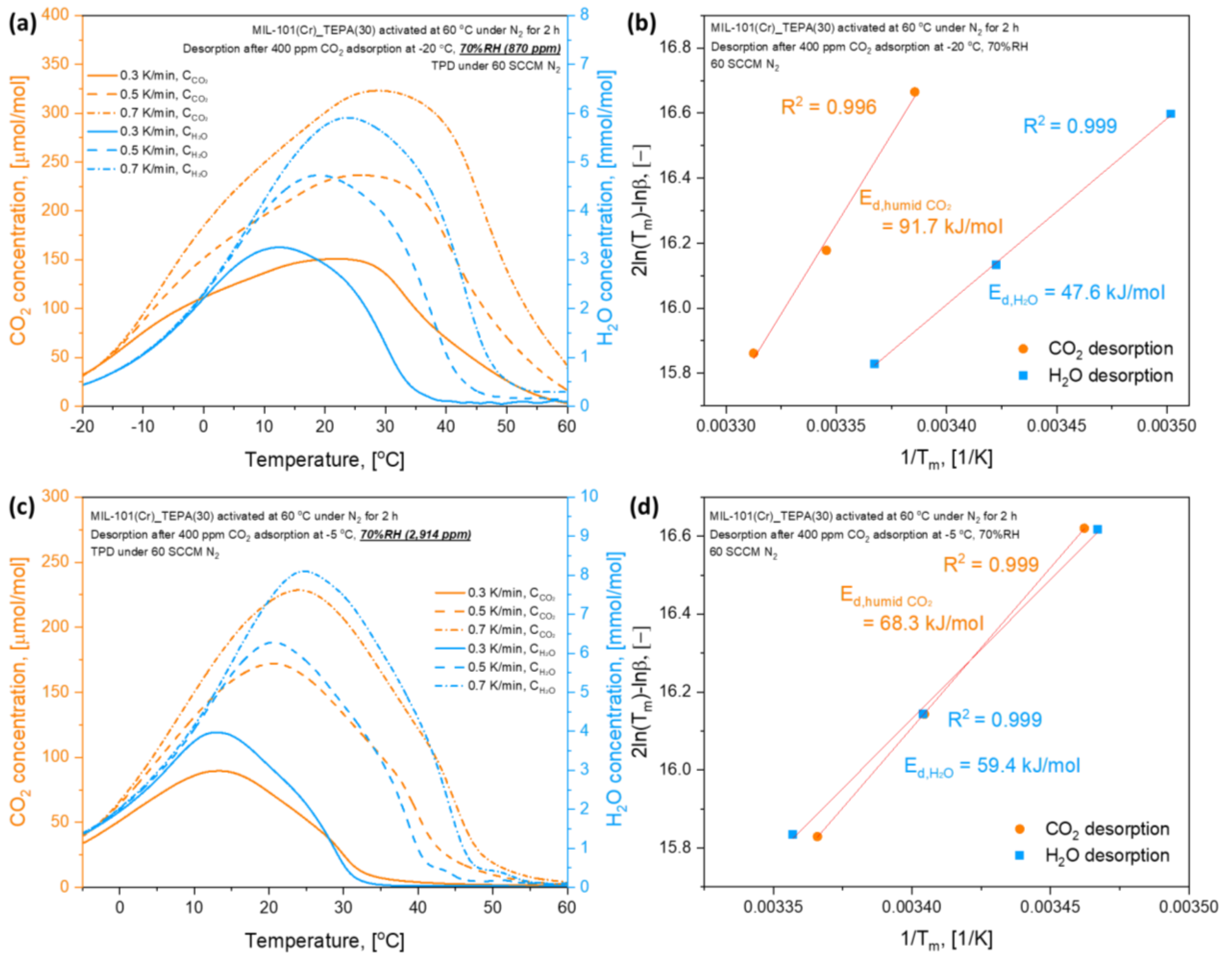

Figure S21. (a/c) CO_2/H_2O TPD profiles of 30 wt% TEPA impregnated MIL-101(Cr) powder sorbents with varied heating rate (0.3, 0.5, 0.7 K/min) and (b/d) determination of the energy of CO_2 and H_2O desorption based on microkinetic analysis for humid (70%RH) 400 ppm CO_2 adsorption at (a/b) -20 ºC and (c/d) -5 ºC. Adsorption conditions: gas, 400 ppm CO_2/N_2; flow rate, 40 sccm.

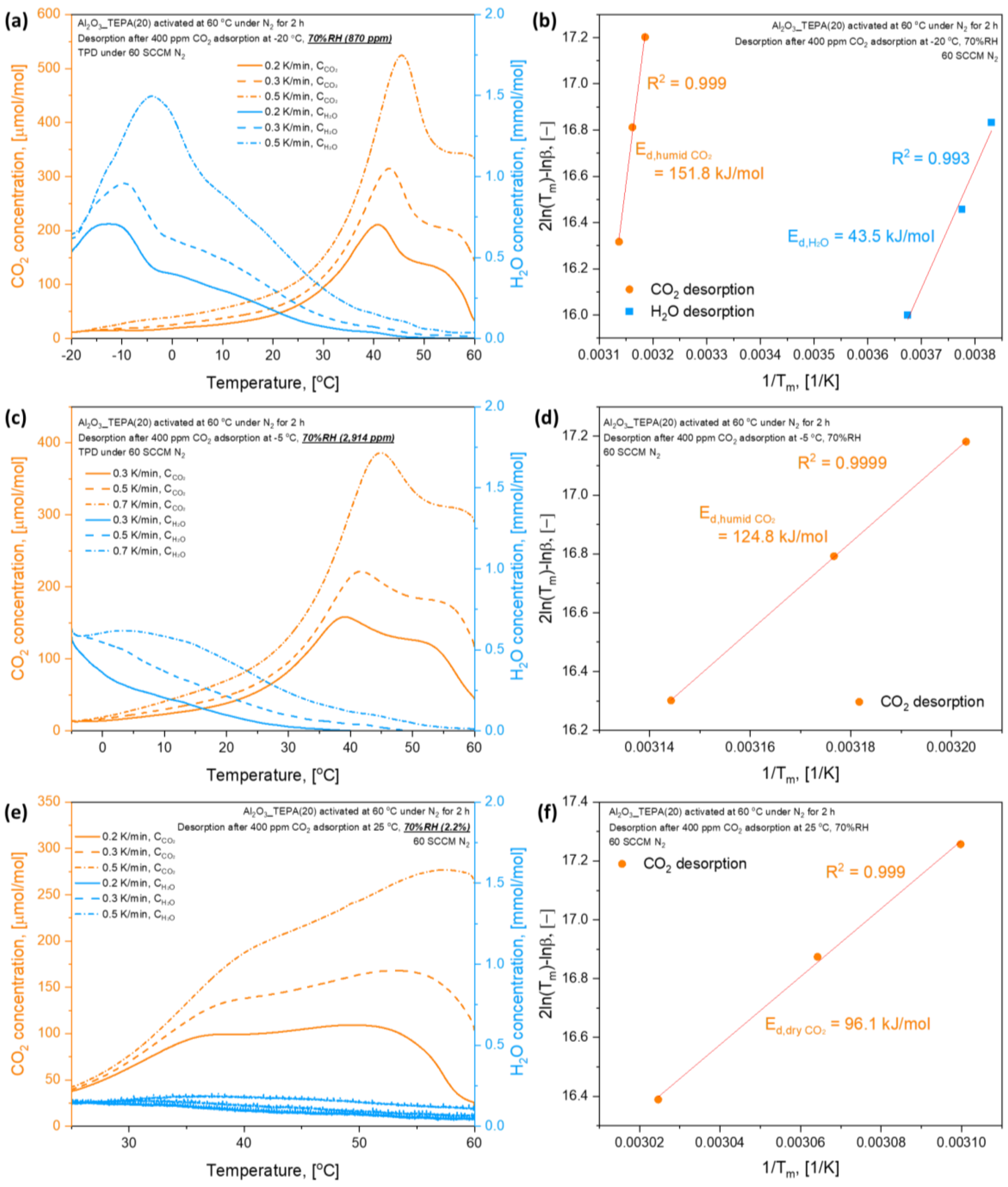

Figure S22. (a/c/e) CO_2/H_2O TPD profiles of 20 wt% TEPA impregnated γ-Al_2O_3 powder sorbents with varied heating rate (0.2, 0.3, 0.5 K/min) and (b/d/f) determination of the energy of CO_2 and H_2O desorption based on microkinetic analysis for humid (70%RH) 400 ppm CO_2 adsorption at (a/b) -20 ºC, (c/d) -5 ºC, and (e/f) 25 ºC. Adsorption conditions: gas, 400 ppm CO_2/N_2; flow rate, 40 sccm.

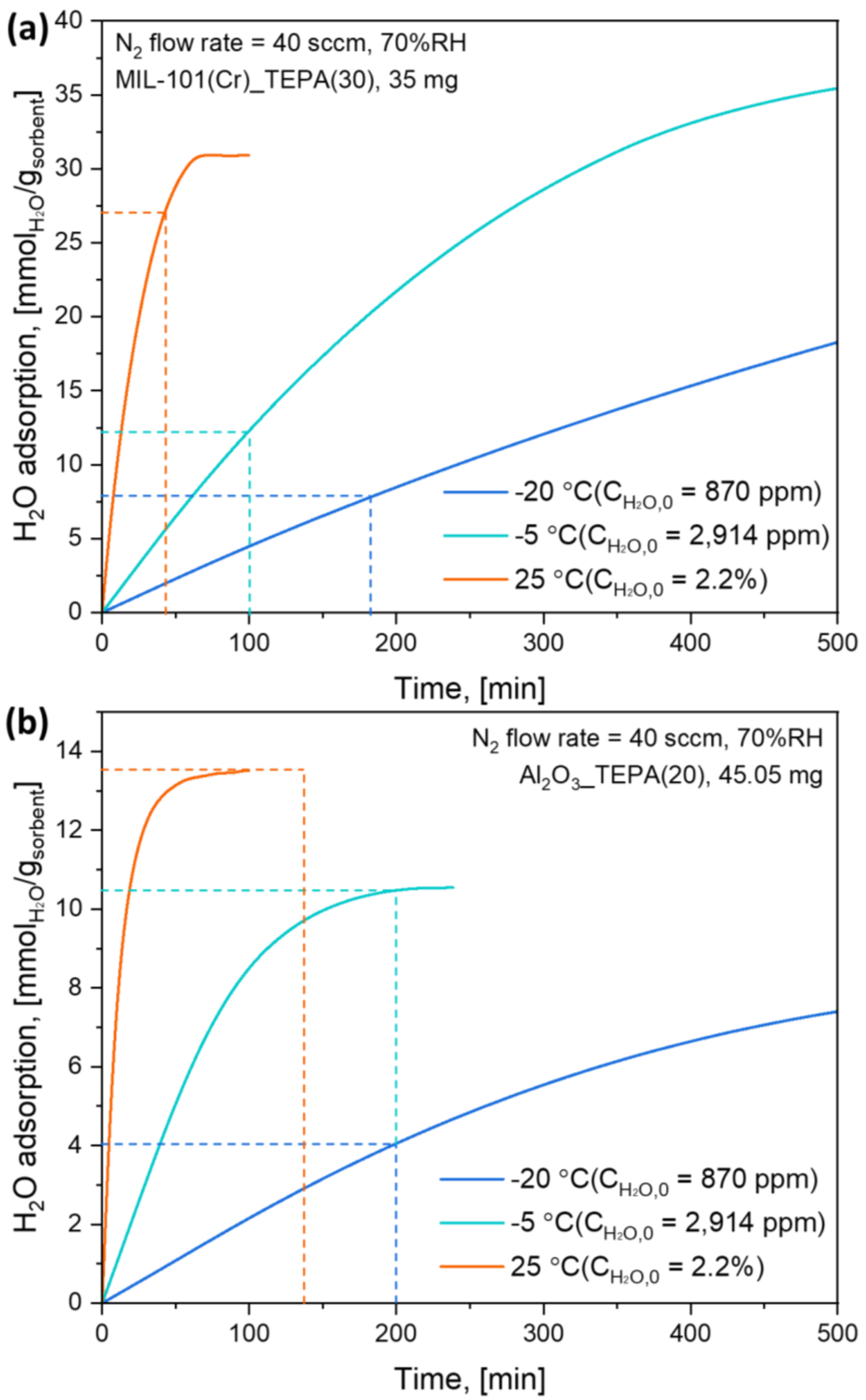

Figure S23. H_2O adsorption curves of (a) 30 wt% TEPA impregnated MIL-101(Cr) and (b) 20 wt% TEPA impregnated γ-Al_2O_3 at -20 °C, -5 °C, and 25 °C. Calculated based on H_2O breakthrough curves shown in **Figure S7**. Adsorption conditions: gas, N_2; flow rate, 40 sccm; relative humidity, 70%RH; activation, 60 ºC under 40 sccm N_2 for 2 - 3 h.

Table S1. FT-IR peak assignments for the CO_2 adsorption.

Frequency, [cm^{-1}]	Assignment	Group	Reference
3470	νOH	adsorbed water	[8, 12-13]
2335	ν_{as}C=O	physisorbed linear CO_2	[4, 14-16]
1700	νC=O	carbamic acid	[4, 6, 15, 17-18]
1658	νC=O	carbamic acid	[17]
1650 - 1635	$\delta_{as}NH^{3+}$	ammonium ion	[4, 8, 11, 14, 16, 18]
1580	$\nu_{as}COO^-$	carbamate ion	[17]
1552	$\nu_{as}COO^-$	carbamate ion	[4, 6, 8, 17-18]
1537	$\delta_{as}NH^{3+}$	ammonium ion	[4, 6, 16-17, 19-20]
1520 – 1510	δNH/νCN	surface bound carbamate	[4, 6, 16]
1488	νCOO^-	carbamate ion	[8, 18, 21]
1473	$\delta_{s}NH^{3+}$	ammonium ion	[17]
1435 - 1425	$\nu_{s}COO^-$	carbamate ion	[4, 6, 14, 16-17, 20, 22]
1410	νCN/$NCOO^-$ skeletal vibration	carbamate ion	[11, 15, 18]
1380 - 1375	$\nu_{s}COO^-$	carbamate ion	[4, 16-17, 20, 22]
1358	$\nu_{s}COO^-$	bicarbonate ion	[6, 17, 23-26]
1320	$NCOO^-$ skeletal vibration	carbamate ion	[6, 8, 11, 14-15, 18, 27-28]

Table S2. DAC performance and the energy for CO_2/H_2O desorption of MIL-101(Cr)_TEPA(30) and Al_2O_3_TEPA(20) sorbent materials (desorption at 25 °C under N_2, small temperature swing).

	**MIL-101(Cr)_TEPA(30)**	**Al_2O_3_TEPA(20)**
Adsorption temperature, [°C]	-20	-20
qCO_2, [mol/kg]	1.55	0.43
qH_2O, [mol/kg]	7.7	4.1
E_{d,CO_2}, [kJ/mol]	91	121.1
E_{d,H_2O}, [kJ/mol]	53.5	46.4
Energy for CO_2 desorption, [GJ/ton CO_2]	2.07	2.75
Energy for H_2O desorption, [GJ/ton CO_2]	6.04	11.6
Sensible heat for sorbent, [GJ/ton CO_2]	0.6	2.0

References

1. Numaguchi, R.; Chowdhury, F. A.; Yamada, H.; Yogo, K., Carbon Dioxide Absorption using Solid Sorbents Incorporating Purified Components of Tetraethylenepentamine. *Energy Technol.* **2017,** *5* (8), 1186-1190.
2. Rim, G.; Kong, F. H.; Song, M. Y.; Rosu, C.; Priyadarshini, P.; Lively, R. P.; Jones, C. W., Sub-Ambient Temperature Direct Air Capture of CO_2 using Amine-Impregnated MIL-101(Cr) Enables Ambient Temperature CO_2 Recovery. *JACS Au* **2022,** *2* (2), 380-393.
3. Cvetanović, R. J.; Amenomiya, Y., Application of a Temperature-Programmed Desorption Technique to Catalyst Studies. In *Adv. Catal.*, Eley, D. D.; Pines, H.; Weisz, P. B., Eds. Academic Press: 1967; Vol. 17, pp 103-149.
4. Bacsik, Z.; Ahlsten, N.; Ziadi, A.; Zhao, G. Y.; Garcia-Bennett, A. E.; Martin-Matute, B.; Hedin, N., Mechanisms and Kinetics for Sorption of CO_2 on Bicontinuous Mesoporous Silica Modified with n-Propylamine. *Langmuir* **2011,** *27* (17), 11118-11128.
5. Hedin, N.; Bacsik, Z., Perspectives on the adsorption of CO_2 on amine-modified silica studied by infrared spectroscopy. *Current Opinion in Green and Sustainable Chemistry* **2019,** *16*, 13-19.
6. Didas, S. A.; Salcwa-Novak, M. A.; Foo, G. S.; Sievers, C.; Jones, C. W., Effect of Amine Surface Coverage on the Co-Adsorption of CO_2 and Water: Spectral Deconvolution of Adsorbed Species. *J. Phys. Chem. Lett.* **2014,** *5* (23), 4194-4200.
7. Yoo, C. J.; Lee, L. C.; Jones, C. W., Probing Intramolecular versus Intermolecular CO_2 Adsorption on Amine-Grafted SBA-15. *Langmuir* **2015,** *31* (49), 13350-13360.
8. Yu, J.; Chuang, S. S. C., The Structure of Adsorbed Species on Immobilized Amines in CO_2 Capture: An in Situ IR Study. *Energy Fuels* **2016,** *30* (9), 7579-7587.
9. Hahn, M. W.; Steib, M.; Jentys, A.; Lercher, J. A., Mechanism and Kinetics of CO_2 Adsorption on Surface Bonded Amines. *J. Phys. Chem. C* **2015,** *119* (8), 4126-4135.
10. Li, K. J.; Kress, J. D.; Mebane, D. S., The Mechanism of CO_2 Adsorption under Dry and Humid Conditions in Mesoporous Silica-Supported Amine Sorbents. *J. Phys. Chem. C* **2016,** *120* (41), 23683-23691.
11. Wang, X. X.; Schwartz, V.; Clark, J. C.; Ma, X. L.; Overbury, S. H.; Xu, X. C.; Song, C. S., Infrared Study of CO_2 Sorption over "Molecular Basket" Sorbent Consisting of Polyethylenimine-Modified Mesoporous Molecular Sieve. *J. Phys. Chem. C* **2009,** *113* (17), 7260-7268.
12. Takeuchi, M.; Martra, G.; Coluccia, S.; Anpo, M., Investigations of the structure of H_2O clusters adsorbed on TiO_2 surfaces by near-infrared absorption spectroscopy. *J. Phys. Chem. B* **2005,** *109* (15), 7387-7391.
13. Vico, S.; Palys, B.; Buess-Herman, C., Hydration of a polysulfone anion-exchange membrane studied by vibrational spectroscopy. *Langmuir* **2003,** *19* (8), 3282-3287.
14. Bacsik, Z.; Atluri, R.; Garcia-Bennett, A. E.; Hedin, N., Temperature-Induced Uptake of CO_2 and Formation of Carbamates in Mesocaged Silica Modified with n-Propylamines. *Langmuir* **2010,** *26* (12), 10013-10024.
15. Srikanth, C. S.; Chuang, S. S. C., Infrared Study of Strongly and Weakly Adsorbed CO_2 on Fresh and Oxidatively Degraded Amine Sorbents. *J. Phys. Chem. C* **2013,** *117* (18), 9196-9205.
16. Danon, A.; Stair, P. C.; Weitz, E., FTIR Study of CO_2 Adsorption on Amine-Grafted SBA-15: Elucidation of Adsorbed Species. *J. Phys. Chem. C* **2011,** *115* (23), 11540-11549.

17. Foo, G. S.; Lee, J. J.; Chen, C. H.; Hayes, S. E.; Sievers, C.; Jones, C. W., Elucidation of Surface Species through in Situ FTIR Spectroscopy of Carbon Dioxide Adsorption on Amine-Grafted SBA-15. *Chemsuschem* **2017,** *10* (1), 266-276.
18. Wilfong, W. C.; Srikanth, C. S.; Chuang, S. S. C., In Situ ATR and DRIFTS Studies of the Nature of Adsorbed CO_2 on Tetraethylenepentamine Films. *ACS Appl. Mater. Interfaces* **2014,** *6* (16), 13617-13626.
19. Bossa, J. B.; Borget, F.; Duvernay, F.; Theule, P.; Chiavassa, T., Formation of neutral methylcarbamic acid ($CH_3NHCOOH$) and methylammonium methylcarbamate [$CH_3NH_3^+$][$CH_3NHCO_2^-$] at low temperature. *J. Phys. Chem. A* **2008,** *112* (23), 5113-5120.
20. Bossa, J. B.; Theule, P.; Duvernay, F.; Borget, F.; Chiavassa, T., Carbamic acid and carbamate formation in NH_3:CO_2 ices-UV irradiation versus thermal processes. *Astron. Astrophys.* **2008,** *492* (3), 719-724.
21. Tumuluri, U.; Isenberg, M.; Tan, C. S.; Chuang, S. S. C., In Situ Infrared Study of the Effect of Amine Density on the Nature of Adsorbed CO_2 on Amine-Functionalized Solid Sorbents. *Langmuir* **2014,** *30* (25), 7405-7413.
22. Knofel, C.; Martin, C.; Hornebecq, V.; Llewellyn, P. L., Study of Carbon Dioxide Adsorption on Mesoporous Aminopropylsilane-Functionalized Silica and Titania Combining Microcalorimetry and in Situ Infrared Spectroscopy. *J. Phys. Chem. C* **2009,** *113* (52), 21726-21734.
23. Lee, J. J.; Chen, C. H.; Shimon, D.; Hayes, S. E.; Sievers, C.; Jones, C. W., Effect of Humidity on the CO_2 Adsorption of Tertiary Amine Grafted SBA-15. *J. Phys. Chem. C* **2017,** *121* (42), 23480-23487.
24. Richner, G.; Puxty, G., Assessing the Chemical Speciation during CO_2 Absorption by Aqueous Amines Using in Situ FTIR. *Ind. Eng. Chem. Res.* **2012,** *51* (44), 14317-14324.
25. Robinson, K.; McCluskey, A.; Attalla, M. I., An FTIR Spectroscopic Study on the Effect of Molecular Structural Variations on the CO_2 Absorption Characteristics of Heterocyclic Amines. *Chemphyschem* **2011,** *12* (6), 1088-1099.
26. Robinson, K.; McCluskey, A.; Attalla, M. I., An ATR-FTIR Study on the Effect of Molecular Structural Variations on the CO_2 Absorption Characteristics of Heterocyclic Amines, Part II. *Chemphyschem* **2012,** *13* (9), 2331-2341.
27. Babin, A.; Vaneeckhaute, C.; Iliuta, M. C., Potential and challenges of bioenergy with carbon capture and storage as a carbon-negative energy source: A review. *Biomass Bioenergy* **2021,** *146*, 105968.
28. Kim, S.; Ida, J.; Guliants, V. V.; Lin, J. Y. S., Tailoring pore properties of MCM-48 silica for selective adsorption of CO_2. *J. Phys. Chem. B* **2005,** *109* (13), 6287-6293.
